# A Rare De Novo Missense Mutation in IFT122 Confers a Genetic Susceptibility Factor of Idiopathic Pediatric Uveitis Via Trio‐based Whole‐Exome Sequencing

**DOI:** 10.1002/advs.76991

**Published:** 2026-09-02

**Authors:** Qian Zhou, Jiaxing Huang, Yilin Wang, Xingran Li, Xianyang Liu, Lingyu Dai, Hongshun Li, Qingfeng Wang, Jiangyi Liu, Guannan Su, Wanyun Zhang, Yang Deng, Qingfeng Cao, Yujie Lai, Xiang Luo, Changwei Huang, Ling Chen, Shengping Hou, Peizeng Yang

**Affiliations:** ^1^ Ophthalmology Medical Center Chongqing Key Laboratory for the Prevention and Treatment of Major Blinding Eye Diseases The First Affiliated Hospital of Chongqing Medical University Chongqing Branch (Municipality Division) of National Clinical Research Centre for Ocular Diseases Chongqing China; ^2^ Beijing Institute of Ophthalmology Beijing Tongren Eye Center Beijing Ophthalmology and Visual Sciences Key Laboratory Beijing Tongren Hospital Capital Medical University Beijing China; ^3^ Department of Ophthalmology Eye and ENT Hospital of Fudan University Shanghai China; ^4^ Department of Ophthalmology Henan International Joint Research Laboratory for Ocular Immunology and Retinal Injury Repair The First Affiliated Hospital of Zhengzhou University Henan Province Eye Hospital Zhengzhou China

**Keywords:** exome sequencing, missense mutation, proteomics, transcription factor, uveitis

## Abstract

Idiopathic pediatric uveitis (IPU) is a leading cause of irreversible vision loss in children; however, the genetic and molecular mechanisms underlying this condition remain unclear. Herein, trio‐based whole‐exome sequencing was performed in 28 affected families and targeted sequencing was performed in 1953 sporadic cases from a Han Chinese cohort. A rare missense mutation, A773E in intraflagellar transport 122 (*IFT122*), was identified in one trio and absent from sporadic cases. Functional assays showed that deleterious IFT122‐A773E increased inflammatory factor secretion and exacerbated barrier function damage both in vivo and in vitro. Further studies using proteomics demonstrated that IFT122‐A773E increased AP‐1 transcription factor subunit (FRA1) expression. The IFT122‐A773E substitution enhanced the interaction with IFT43 and up‐regulated calcium channels, and in turn led to activation of the MEK/ERK signaling axis. Collectively, our findings suggest that IFT122‐A773E may increase susceptibility to IPU through activation of the MEK/ERK/FRA1 axis.

## Graphical abstract

A rare de novo IFT122‐A773E variant is identified in idiopathic pediatric uveitis and shown to exacerbate retinal inflammation and barrier dysfunction. Mechanistically, the variant enhances IFT43 interaction, elevates calcium signaling, and activates the MEK/ERK/FRA1 axis, revealing a previously unrecognized cilia‐associated pathway that may increase disease susceptibility.

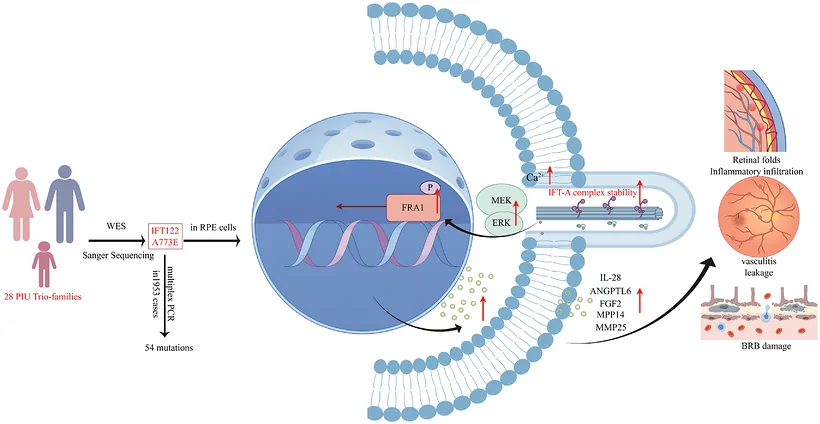

## 1 Introduction

Uveitis encompasses a group of inflammatory disorders that affect the uveal tract and adjacent ocular structures, such as the vitreous, retina, and optic nerve [1, 2]. Recurrent inflammation often causes irreversible damage to the retina and optic nerve, resulting in permanent vision loss. Epidemiological studies have indicated that uveitis contributes to > 25% of visual impairment cases in developing countries [2].

Pediatric uveitis accounts for approximately 5%–10% of all uveitis cases, with autoimmune etiologies representing 67.2%–93.8% of pediatric cases. Compared with adult‐onset disease, pediatric uveitis often follows a chronic and asymptomatic course, frequently resulting in progressive ocular structural damage [3]. Delayed diagnosis is common in children because of subtle clinical manifestations and challenges associated with ophthalmic examination [4, 5]. Additionally, pediatric uveitis is often persistent, recurrent, and resistant to conventional therapies. Although some cases are associated with systemic autoimmune diseases, such as juvenile idiopathic arthritis, juvenile sarcoidosis (Blau syndrome), and Behçet's disease, a substantial proportion of pediatric uveitis cases lack identifiable systemic or infectious causes and are therefore classified as idiopathic pediatric uveitis (IPU). Notably, IPU has been reported to account for 71.3% of all pediatric cases in a Chinese cohort [6, 7, 8]. Therefore, the development of novel diagnostic and therapeutic approaches is crucial.

Autoimmune uveitis, including IPU, is widely recognized as resulting from a complex interaction between genetic susceptibility and environmental triggers [9, 10]. To date, no distinct Mendelian inheritance pattern has been identified in IPU; however, familial clustering in certain uveitis cases implies potential genetic susceptibility [11]. Advances in high‐throughput sequencing have enabled the identification of gene loci, such as small ubiquitin‐like modifier 4, monocyte chemoattractant protein‐1, interleukin‐17 (IL‐17), and cytotoxic T‐lymphocyte‐associated protein 4, in adult uveitis, particularly in Behçet's disease and Vogt‐Koyanagi‐Harada syndrome [12, 13, 14, 15]. However, the genetic mechanisms underlying IPU remain unclear. Identifying susceptibility genes for IPU may provide novel insights into disease mechanisms and support precision treatment strategies.

Experimental autoimmune uveitis (EAU), a widely used mouse model, exhibits clinical manifestations and pathological alterations similar to those observed in human autoimmune uveitis and is, therefore, extensively used to investigate disease mechanisms and susceptibility factors [16, 17, 18]. EAU model mice are immunized with an adjuvant containing conserved retinal antigens that are processed and presented to naïve T‐cells by antigen‐presenting cells. Subsequently, naïve T‐cells differentiate into effector T‐cells, which secrete inflammatory mediators and promote breakdown of the blood‐retinal barrier (BRB). Once the BRB is compromised, circulating immune cells and proinflammatory molecules infiltrate the retina, exacerbating local immune activation and contributing to retinal damage [19, 20].

Located in the outer retina, the retinal pigment epithelium (RPE) plays a crucial role in retinal cellular interactions and immune regulation. As a critical component of the BRB, the RPE regulates metabolic exchange, scavenges reactive oxygen species (ROS), and maintains the stability of the retinal microenvironment [21, 22, 23, 24]. RPE damage is closely associated with autoimmune uveitis. For example, mutations in MPP2 and ORH11 in the RPE cells increased the susceptibility to Vogt‐Koyanagi‐Harada disease, whereas METTL3 expression in RPE can attenuate inflammation in autoimmune uveitis [20, 25, 26]. These findings indicated that functional abnormalities in the RPE contribute to the development of autoimmune uveitis.

In the present study, we aimed to identify genetic variants associated with IPU and further explore their potential functional roles and molecular mechanisms, thereby providing a theoretical foundation for elucidating IPU pathogenesis and paving the way for future precision and personalized therapies. Therefore, trio‐based whole‐exome sequencing (WES) was performed in 28 patients and their unaffected biological parents, along with sequencing of 1953 sporadic IPU cases. Our findings may provide potential therapeutic targets for IPU.

## 2 Results

### 2.1 A Rare De Novo Mutation IFT122 p.A773E is Identified in IPU

**FIGURE 1:**
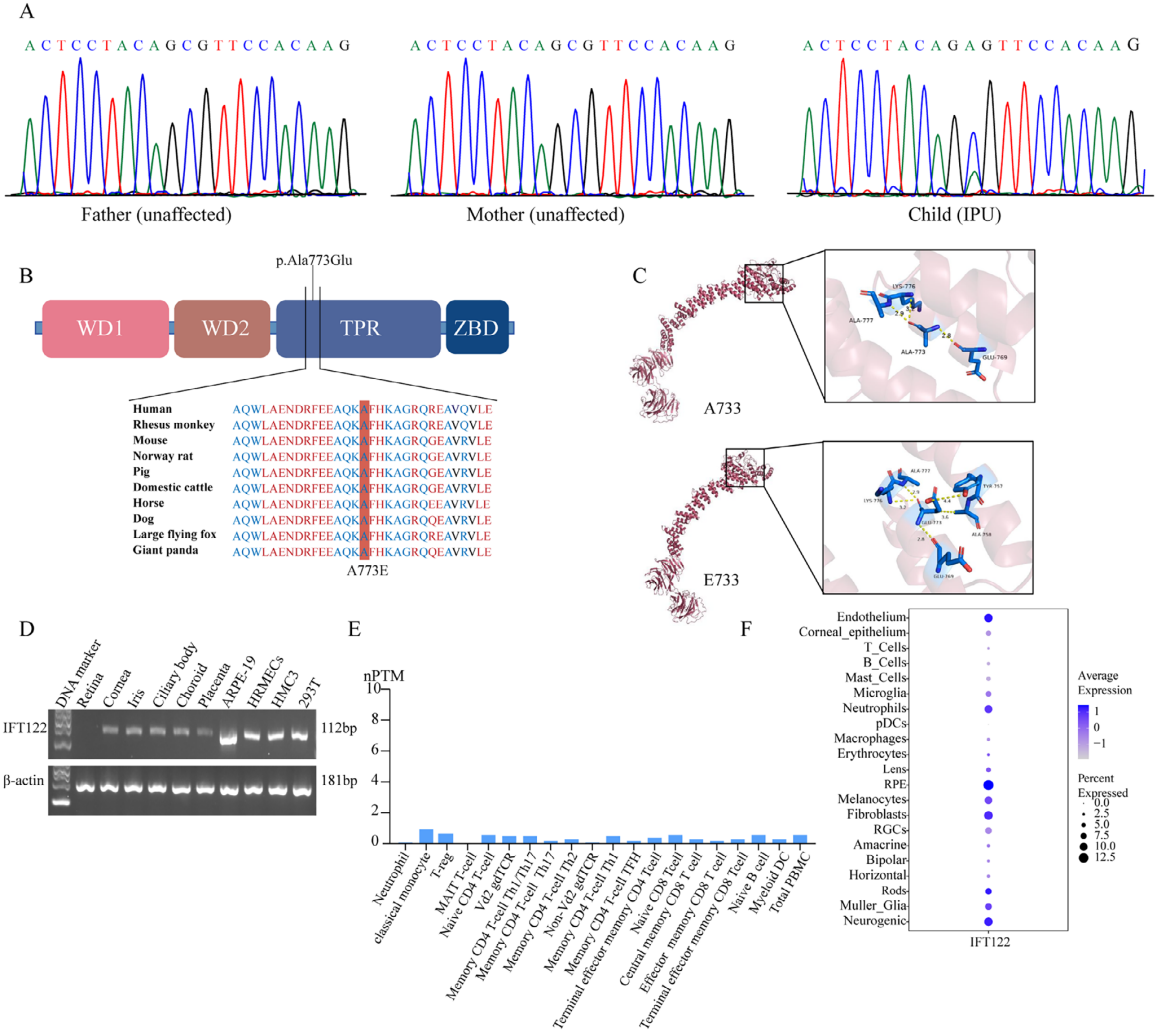
Identification of de novo mutations of IPU. (A) Identification of the de novo IFT122 p.A773 E mutation in one IPU trio family. (B) The predicted conservation for the IFT122‐A773E mutation in humans, Rhesus monkeys, mouse, etc. (C) The structure prediction of the IFT122‐A773 and IFT122‐E773 proteins using AlphaFold. (D). The expression profile of the IFT122 gene in several human tissues and cell lines. (E) The nTPM of IFT122 in human immune cells. (F) The expression levels of IFT122 in human eye tissue, including RPE, endothelium, immune cells, etc.

To assess the genetic basis of IPU, we performed WES in 28 children with IPU (mean age: 5.25 years; range: 2–10 years) and their 56 unaffected parents of Han Chinese ancestry. We identified 33 de novo variants in this cohort (Table 1). Among these variants, we prioritized an unreported missense mutation in IFT122, resulting in an alanine‐to‐glutamic acid substitution at codon 773 (p. Ala773Glu; c.2318C>A; NM_052990) in exon 19 on chromosome 3. This variant was detected in a single trio and was further confirmed using Sanger sequencing (Figure 1A). Quantitative reverse‐transcription PCR (RT‐qPCR) analysis revealed that IFT122 transcript levels did not differ significantly between the p.A773E variant and wild‐type (WT) alleles, indicating that the mutation does not affect mRNA expression (Figure S1A).

**TABLE 1:** The Summary of de novo mutations in 28 PIU trios families.

GeneName	Chr	Pos	Ref	Alt	AAchange	ExonicFunc
ABCA13	7	48559870	G	A	NM_152701:c.G14031A:p.W4677X	stopgain
OR1N2	9	125316325	G	T	NM_001004457:c.G877T:p.V293F	missense SNV
CYTIP	2	158300367	C	T	NM_004288:c.C166T:p.R56X	stopgain ‐
NUTM2A	10	88992455	C	T	NM_001099338:c.C1447T:p.P483S	missense SNV
WDR46	6	33248760	A	G	NM_001164267:c.A958G:p.M320V	missense SNV
DLX4	17	48051060	C	T	/	/
NOLC1	10	103912169	T	C	NM_001284388:c.T2C:p.M1T	missense SNV
TRIM39	6	30303682	G	T	NM_001199119:c.G710T:p.R237L	missense SNV
ZDHHC11	5	840712	G	C	NM_024786:c.G682C:p.V228L	missense SNV
KCNC3	19	50824026	G	C	NM_004977:c.G1994C:p.G665A	missense SNV
PLCD3	17	43192557	G	C	/	/
SLC6A20	3	45812857	G	A	NM_022405:c.G676A:p.A226T	missense SNV
CCDC74B	2	130899886	G	C	NM_207310:c.G364C:p.G122R	missense SNV
KIF26A	14	104641549	C	G	NM_015656:c.C2424G:p.N808K	missense SNV
RPIA	2	89049521	A	G	NM_144563:c.A862G:p.I288V	missense SNV
FOXA1	14	38060595	C	G	NM_004496:c.C1394G:p.S465C	missense SNV
KRTAP1‐3	17	39190973	G	C	NM_030966:c.G101C:p.C34S	missense SNV
IFT122	3	129225252	C	A	NM_052990: c.C2318A:p.A773E,	missense SNV
SLC25A33	1	9627364	C	G	NM_032315:c.C259G:p.P87A	missense SNV
QRICH2	17	74288410	G	A	NM_032134:c.G1900A:p.G634S	missense SNV
KRT6C	12	52865970	G	A	NM_173086:c.C635T:p.P212L	missense SNV
ZNFX1	20	47865437	C	G	NM_021035:c.C4124G:p.P1375R	missense SNV
WNK4	17	40933072	C	T	NM_032387:c.C356T:p.A119V	missense SNV
CYP4A11	1	47399915	C	T	NM_000778:c.C1021T:p.P341S	missense SNV
RASA2	3	141327470	A	G	NM_001303245:c.A2159G:p.Y720C	missense SNV
RHAG	6	49585876	C	G	NM_000324:c.C397G:p.L133V	missense SNV
LOC100129697	16	89017502	C	G	NM_001290330:c.C976G:p.P326A	missense SNV
TCP11	6	35088683	T	C	/	/
RAB40C	16	677488	G	A	NM_021168:c.G712A:p.G238S	missense SNV
RPL29	3	52027873	C	CT	NM_000992:c.371dupA:p.K124fs	frameshift insertion
ZNF480	19	52803668	GCT	G	NM_001297624:c.4_5del:p.L2fs	frameshift deletion
CCDC40	17	78063947	/	A	NM_001243342:c.2843_2874del:p.R948fs	frameshift deletion
OR2T8	1	248084470	C	CG	NM_001005522:c.151_152insG:p.H51fs	frameshift insertion
SLCO1B1	12	21375305	/	T	/	/
AKNAD1	1	109377154	AT	A	/	/
MST1L	1	17085989	ACGCC	A	NM_001271733:c.904_907del:p.G302fs	frameshift deletion
MST1L	1	17085992	CCCG	C	NM_001271733:c.902_904del:p.301_302del	nonframeshift deletion
FMN2	1	240255562	GGGA	G	NM_001305424:c.154_156del:p.52_52del	nonframeshift deletion
ARHGEF5	7	144059768	G	/	NM_005435:c.6_7insGCTGAGGAGGCCCAGCGTGGAA:p.E2fs	frameshift insertion
MST1L	1	17085991	GCCCGC	G	NM_001271733:c.901_905del:p.A301fs	frameshift deletion

We identified no prior reports of this variant in IPU. The variant is absent from large‐scale population databases, including the 1000 Genomes Project (1000 g_all), Exome Sequencing Project (ESP6500), and Genome Aggregation Database (gnomAD_ALL and gnomAD_EAS), indicating that it is extremely rare and potentially disease‐associated. The IFT122 p.A773E variant occurs at a highly conserved residue across multiple vertebrate species, including humans, monkeys, mice, rats, pigs, cattle, horses, dogs, bats, and giant pandas, highlighting its evolutionary conservation (Figure 1B). Using multiple bioinformatic tools, we predicted that the A773E mutation is deleterious and impairs normal protein function (Table 2). Structural modeling using AlphaFold further indicated that A773E induces significant conformational alterations in IFT122 (Figure 1C). Together, these findings suggested that the IFT122 p.A773E variant is potentially pathogenic.

**TABLE 2:** The deleteriousness of IFT122‐A773E mutation by predicted software.

IFT122	SIFT	Polyphen2_HVAR	Polyphen2_HDIV	Mutation Taster	CADD	gerp++gt2	MCAP
A773E	0.001,D	1.0,D	1.0,D	1,D	7.215251,34	5.62	0.319418

Subsequently, we performed multiplex PCR sequencing of *IFT122* coding regions in an independent cohort of 1953 individuals with IPU and identified 54 missense variants (Table 3). The pathogenicity of these variants was further assessed using in silico prediction algorithms and population‐level frequency annotations (Table 4). Although we detected several variants, such as p.R679Q, in multiple individuals, most of them were predicted to be tolerated or occurred in the general population databases, with limited evidence supporting their pathogenicity. The de novo A773E variant, identified by trio‐based WES, was absent from both the validation cohort and general population databases, and was consistently predicted to be deleterious by multiple in silico tools, supporting its prioritization for functional studies. Expression analysis revealed that IFT122 is expressed in ocular tissues, including the ciliary body, iris, choroid, and retina, as well as in cultured human cell lines, such as ARPE19, HUVECs, and HEK293T cells. ARPE19 cells exhibited the highest IFT122 expression (Figure 1D). According to Human Protein Atlas data, IFT122 expression was low in immune cells, including T‐cells, B‐cells, monocytes, dendritic cells, and natural killer cells (Figure 1E). Analysis of our previous single‐cell RNA sequencing dataset of the fetal retina showed that IFT122 expression is enriched in human RPE cells, whereas its expression in immune cells is lower (Figure 1F) [27]. These results suggested that IFT122 may contribute to IPU pathogenesis through modulation of RPE function.

**TABLE 3:** The IFT122 mutations in 1954 PIU patients by multiplex PCR.

Chr	Pos/Start	Ref	Alt	AAchange	Frequency	ExonicFunc
3	129214302	G	A	NM_001280541: c.G2036A:p.R679Q	241/1954	nonsynonymous SNV
3	129195166	G	T	NM_001280541: c.G801T:p.K267N	117/1954	nonsynonymous SNV
3	129195600	G	A	NM_001280541: c.G1079A:p.S360N	68/1954	nonsynonymous SNV
3	129238472	G	A	NM_001280541: c.G3512A:p.R1171H	30/1954	nonsynonymous SNV
3	129207210	AGA	del_AGA	NM_001280541:c.1938_1940del:p.646_647del	19/1954	nonframeshift deletion
3	129185806	G	A	NM_052985: c.G790A:p.E264K	11/1954	nonsynonymous SNV
3	129207191	A	C	NM_001280541: c.A1919C:p.E640A	5/1954	nonsynonymous SNV
3	129223243	C	T	NM_001280541: c.C2605T:p.R869C	4/1954	nonsynonymous SNV
3	129225282	C	T	NM_001280541: c.C2657T:p.A886V	3/1954	nonsynonymous SNV
3	129183597	T	G	NM_001280541: c.T689G:p.I230R	2/1954	nonsynonymous SNV
3	129195171	G	A	NM_001280541: c.G806A:p.R269Q	2/1954	nonsynonymous SNV
3	129200460	C	T	NM_001280541: c.C1552T:p.R518C	2/1954	nonsynonymous SNV
3	129214389	G	T	NM_001280541: c.G2123T:p.S708I	2/1954	nonsynonymous SNV
3	129221676	A	T	NM_001280541: c.A2474T:p.D825V	2/1954	nonsynonymous SNV
3	129223183	C	A	NM_001280541: c.C2545A:p.H849N	2/1954	nonsynonymous SNV
3	129159200	T	G	NM_001280541: c.T27G:p.D9E	1/1954	nonsynonymous SNV
3	129179827	G	A	NM_001280541: c.G403A:p.V135I	1/1954	nonsynonymous SNV
3	129183479	T	A	NM_001280541: c.T571A:p.W191R	1/1954	nonsynonymous SNV
3	129185735	/	/	NM_052985: c.719_753del:p.R240fs	1/1954	frameshift deletion
3	129185753	T	A	NM_052985: c.T737A:p.M246K	1/1954	nonsynonymous SNV
3	129185780	A	G	NM_052985: c.A764G:p.D255G	1/1954	nonsynonymous SNV
3	129185792	A	G	NM_052985: c.A776G:p.N259S	1/1954	nonsynonymous SNV
3	129195244	C	G	NM_001280541: c.C879G:p.D293E	1/1954	nonsynonymous SNV
3	129195278	C	T	NM_001280541: c.C913T:p.R305W	1/1954	nonsynonymous SNV
3	129195318	C	T	NM_001280541: c.C953T:p.T318M	1/1954	nonsynonymous SNV
3	129195541	C	A	NM_001280541: c.C1020A:p.Y340X	1/1954	nonsynonymous SNV
3	129195570	T	C	NM_001280541: c.T1049C:p.L350P	1/1954	nonsynonymous SNV
3	129195586	T	ins_T	NM_001280541: c.1065dupT:p.Y355fs	1/1954	frameshift insertion
3	129196891	A	G	NM_001280541: c.A1156G:p.I386V	1/1954	nonsynonymous SNV
3	129196940	T	G	NM_001280541: c.T1205G:p.L402R	1/1954	nonsynonymous SNV
3	129196943	T	C	NM_001280541: c.T1208C:p.I403T	1/1954	nonsynonymous SNV
3	129198643	T	C	NM_001280541: c.T1342C:p.C448R	1/1954	nonsynonymous SNV
3	129198657	C	G	NM_001280541: c.C1356G:p.S452R	1/1954	nonsynonymous SNV
3	129198658	G	A	NM_001280541: c.G1357A:p.G453R	1/1954	nonsynonymous SNV
3	129198734	A	G	NM_001280541: c.A1433G:p.E478G	1/1954	nonsynonymous SNV
3	129200518	C	T	NM_001280541: c.C1610T:p.T537I	1/1954	nonsynonymous SNV
3	129202341	A	G	NM_001280541: c.A1643G:p.N548S	1/1954	nonsynonymous SNV
3	129202362	A	G	NM_001280541: c.A1664G:p.Q555R	1/1954	nonsynonymous SNV
3	129202478	A	G	NM_001280541: c.A1780G:p.I594V	1/1954	nonsynonymous SNV
3	129207106	C	T	NM_001280541: c.C1834T:p.P612S	1/1954	nonsynonymous SNV
3	129221660	T	C	NM_001280541: c.T2458C:p.Y820H	1/1954	nonsynonymous SNV
3	129221676	A	C	NM_001280541: c.A2474C:p.D825A	1/1954	nonsynonymous SNV
3	129225311	A	G	NM_001280541: c.A2686G:p.N896D	1/1954	nonsynonymous SNV
3	129233272	G	A	NM_001280541: c.G3007A:p.G1003S	1/1954	nonsynonymous SNV
3	129233300	A	G	NM_001280541: c.A3035G:p.Y1012C	1/1954	nonsynonymous SNV
3	129233302	G	A	NM_001280541: c.G3037A:p.D1013N	1/1954	nonsynonymous SNV
3	129233312	G	A	NM_001280541: c.G3047A:p.R1016H	1/1954	nonsynonymous SNV
3	129233329	G	T	NM_001280541: c.G3064T:p.A1022S	1/1954	nonsynonymous SNV
3	129234371	C	T	NM_001280541: c.C3173T:p.P1058L	1/1954	nonsynonymous SNV
3	129234410	G	A	NM_001280541: c.G3212A:p.R1071H	1/1954	nonsynonymous SNV
3	129236405	G	A	NM_001280541: c.G3338A:p.R1113Q	1/1954	nonsynonymous SNV
3	129237991	G	A	NM_001280541: c.G3412A:p.G1138R	1/1954	nonsynonymous SNV
3	129238448	G	A	NM_001280541: c.G3488A:p.R1163Q	1/1954	nonsynonymous SNV
3	129238471	C	T	NM_001280541: c.C3511T:p.R1171C	1/1954	nonsynonymous SNV

**TABLE 4:** Database annotation and pathogenicity prediction of IFT122 variants.

AAchange	AVSNP150	ESP65000	1000Genome	1000Genome‐Eas	SIFT	PP2_HDIV	PP2_HVAR	MT	FATHMM	PROVEAN	MCAP	CADD	GERP
R679Q	rs61740161	0.0787	.	0.0602	0.209,T	0.068,B	0.008,B	0.001,P	−1,P	0.431,N	.	0.497	0.375
K267N	rs117517364	.	0.00559105	0.0387	0.002,D	0.441,B	0.261,B	1,D	−2.7,D	0.797,D	.	0.666	0.63
S360N	rs150550701	0.0012	0.00559105	0.013	1,T	0.005,B	0.009,B	1,D	0.44,T	0.007,N	.	0.163	0.603
R1171H	rs149029829	.	0.00159744	0.0083	0.056,T	1,D	0.996,D	1,D	0.21,T	0.513,N	0.063	0.914	0.934
646_647del	.	.	.	.	.	.	.	.	.	.	.	.	
E264K	.	.	.	.	0.484,T	0,B	0,B	1,D	0.39,T	0.07,N	0.018	0.243	0.438
E640A	rs144397126	0.0113	0.00638978	0.0011	0.724,T	0.136,B	0.068,B	1,D	−1.66,D	0.208,N	.	0.256	0.593
R869C	rs773080594	.	.	0.0005	0,D	0.999,D	0.855,P	1,D	−0.07,T	0.922,D	0.076	0.947	0.793
A886V	rs376549217	7.7e‐05	.	0.0016	0.002,D	1,D	0.994,D	1,D	−0.5,T	0.701,D	0.281	0.943	0.857
I230R	rs769527595	.	.	5.437e‐05	0.001,D	0.731,P	0.323,B	0.65,D	−2.88,D	0.898,D	0.381	0.544	0.793
R269Q	rs561707472	.	0.000599042	0.0001	0.011,D	0.846,P	0.163,B	1,D	−2.63,D	0.62,D	0.068	0.524	0.63
R518C	rs149578956	0.0002	0.000199681	5.46e‐05	0,D	1,D	0.897,P	1,D	−2.67,D	0.933,D	0.459	0.953	0.813
S708I	.	.	.	.	0.01,D	0.773,P	0.232,B	1,D	−2.56,D	0.635,D	0.075	0.619	0.743
D825V	rs201218755	.	.	0.0001	0,D	1,D	0.997,D	1,D	0.02,T	0.945,D	0.304	0.873	0.997
H849N	rs780370898	.	.	0.0002	0.394,T	0.009,B	0.009,B	1,D	0.19,T	0.765,D	0.009	0.412	0.793
D9E	.	.	.	0.0004	0.249,T	0.994,D	0.97,D	0.887,D	0.71,T	0.498,N	0.046	0.425	0.39
V135I	rs146653997	7.7e‐05	.	0	0.644,T	0,B	0,B	1,N	0.16,T	0.066,N	0.004	0.078	0.149
W191R	.	.	.	.	0,D	0.997,D	0.99,D	1,D	−0.43,T	0.998,D	0.251	0.828	0.746
R240fs	.	.	.	.	.	.	.	.	.	.	.	.	.
M246K	rs189000680	.	0.000199681	5.437e‐05	0.243,T	0.039,B	0.018,B	1,D	0.5,T	0.103,N	0.028	0.306	0.3
D255G	.	.	.	.	0.021,D	0.048,B	0.043,B	0.887,D	0.3,T	0.136,N	0.04	0.384	0.596
N259S	.	.	.	.	0.322,T	0.001,B	0.003,B	1,D	0.42,T	0.102,N	0.01	0.057	0.111
D293E	.	.	.	.	0.015,D	0.986,D	0.718,P	1,D	−2.97,D	0.488,N	0.097	0.726	0.449
R305W	rs755493101	.	.	0	0.042,D	1,D	0.932,D	1,D	−2.57,D	0.794,D	0.231	0.927	0.904
T318M	.	.	.	.	0.005,D	1,D	0.966,D	1,D	0.17,T	0.559,N	0.148	0.903	0.904
Y340X	.	.	.	0.0001	.	.	.	1,A	.	.	.	0.971	0.561
L350P	.	.	.	.	0,D	1,D	0.999,D	1,D	−2.63,D	0.929,D	0.553	0.806	0.868
Y355fs	.	.	.	.	.	.	.	.	.	.	.	.	.
I386V	.	.	.	.	0.034,D	0.948,P	0.949,D	1,D	−0.97,T	0.234,N	0.038	0.5	0.828
L402R	.	.	.	.	0.007,D	0.631,P	0.428,B	1,D	−0.87,T	0.762,D	0.122	0.716	0.803
I403T	.	.	.	5.437e‐05	0.001,D	0.996,D	0.99,D	1,D	−1.02,T	0.777,D	0.175	0.685	0.803
C448R	.	.	.	.	0.011,D	0.96,D	0.748,P	1,D	−1.08,T	0.91,D	0.171	0.812	0.759
S452R	.	.	.	.	0.324,T	0.001,B	0.003,B	0.547,D	−2.71,D	0.385,N	0.217	0.202	0.023
G453R	rs368786226	7.7e‐05	.	5.437e‐05	0.001,D	0.999,D	0.929,D	1,D	−2.79,D	0.945,D	0.239	0.936	0.759
E478G	.	.	.	5.437e‐05	0.002,D	1,D	0.994,D	1,D	−2.58,D	0.934,D	0.629	0.841	0.716
T537I	.	.	.	.	0.002,D	0.98,D	0.773,P	1,D	−2.93,D	0.834,D	0.529	0.833	0.589
N548S	.	.	.	.	0.055,T	0.998,D	0.987,D	1,D	−2.61,D	0.704,D	0.212	0.526	0.914
Q555R	.	.	.	.	0.117,T	0.992,D	0.979,D	1,D	1.58,T	0.478,N	0.037	0.389	0.868
I594V	.	.	.	.	0.426,T	0.948,P	0.949,D	1,D	1.72,T	0.183,N	0.02	0.253	0.868
P612S	.	.	.	.	0.565,T	1,D	0.998,D	1,D	−0.96,T	0.574,N	0.174	0.372	0.555
Y820H	.	.	.	0.0002	0,D	0.996,D	0.919,D	1,D	−0.03,T	0.753,D	0.164	0.875	0.997
D825A	rs201218755	.	0.000399361	0.0004	0,D	1,D	0.998,D	1,D	−0.03,T	0.969,D	0.338	0.891	0.997
N896D	.	.	.	.	0.01,D	0.998,D	0.991,D	1,D	0.09,T	0.766,D	0.108	0.77	0.857
G1003S	rs747929259	.	.	5.44e‐05	0.008,D	1,D	0.999,D	1,D	0.09,T	0.823,D	0.16	0.95	0.753
Y1012C	rs553449191	.	0.000199681	0	0.06,T	0.023,B	0.021,B	1,D	0.15,T	0.769,D	0.052	0.396	0.753
D1013N	.	.	.	.	0.323,T	0.001,B	0.002,B	1,D	0.21,T	0.522,N	0.035	0.467	0.532
R1016H	rs753932809	.	.	5.44e‐05	0.046,D	0.352,B	0.091,B	0.864,D	0.37,T	0.352,N	0.043	0.426	0.214
A1022S	.	.	.	.	0.352,T	0.001,B	0.001,B	1,N	0.4,T	0.158,N	0.01	0.145	0.217
P1058L	rs368574590	7.7e‐05	.	0.0002	0.005,D	1,D	0.999,D	1,D	−0.06,T	0.963,D	0.205	0.946	0.719
R1071H	.	.	.	.	0.236,T	0.999,D	0.897,P	1,D	0.33,T	0.377,N	0.051	0.532	0.719
R1113Q	rs746890917	.	.	0	0.298,T	0.001,B	0.001,B	1,N	0.28,T	0.255,N	0.024	0.154	0.157
G1138R	.	.	.	.	0.16,T	0.003,B	0.003,B	1,N	0.42,T	0.221,N	0.051	0.231	0.344
R1163Q	rs760157152	.	.	0	0.005,D	0.999,D	0.99,D	1,D	0.15,T	0.657,D	0.171	0.892	0.934
R1171C	rs549826483	.	0.000399361	0	0.012,D	1,D	0.997,D	1,D	0.18,T	0.727,D	0.075	0.957	0.632

*AVSNP150* dbSNP Build 150 Identifier, *ESP6500* Exome Sequencing Project 6500, *1000Genome* 1000 Genomes Project, *1000Genome‐EAS* East Asian population from the 1000 Genomes Project.

Collectively, we identified IFT122‐A773E as a rare de novo mutation associated with IPU. By altering RPE function, this variant may contribute to IPU progression.

### 2.2 IFT122‐A773E Exacerbates EAU and Disrupts the BRB

**FIGURE 2:**
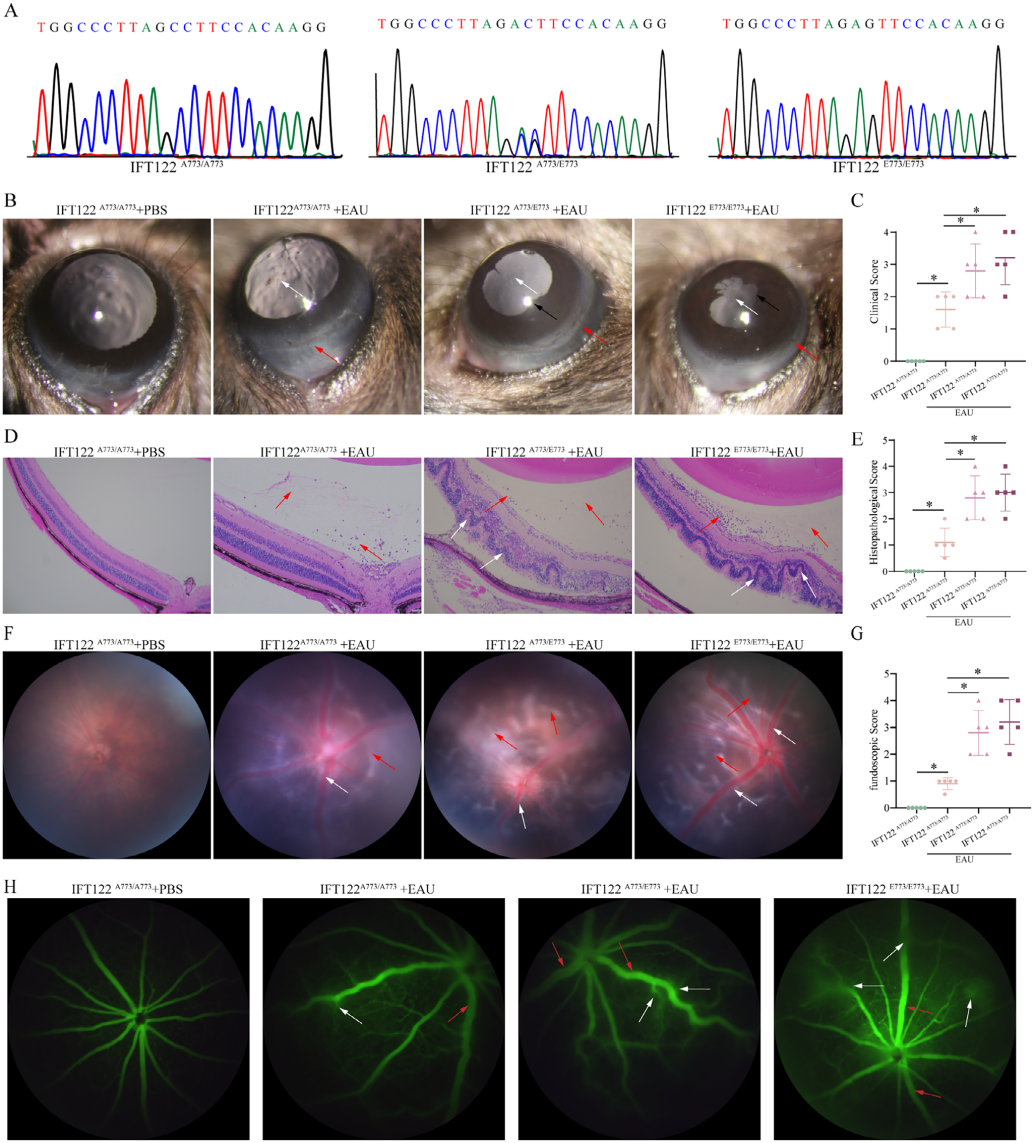
Aggravation of inflammatory response and destruction of the BRB in IFT122‐A773 E mutant mice with EAU. (A) Sanger sequencing of the de novo IFT122 p.A773 E mutation in the mice, including wild type (IFT122^A773/A773^), heterozygotes (IFT122^A773/E773^) and homozygotes (IFT122^E773/E773^). (B,C) Anterior segment inflammation and clinical scores of mice in wild type treated with PBS, and wild type, heterozygotes, and homozygotes adopted EAU. White arrow, corneal opacity, exudate. Red arrow, ciliary hyperemia. Black arrow, irregular pupils and posterior synechiae (n = 5‐8/group; mean ± SD; NS p > 0.05; Kruskal–Wallis test, followed by pairwise Mann–Whitney U tests with Bonferroni correction). (D,E) Hematoxylin‐eosin staining and pathological scores of mice in wild type treated with PBS, and wild type, heterozygotes and homozygotes adopted EAU. Red arrow, inflammatory cells and exudate. White arrow, retina fold. (n = 5‐8/group; mean ± SD; NS p > 0.05, ^*^ *p* < 0.05; Kruskal–Wallis test, followed by pairwise Mann–Whitney U tests with Bonferroni correction). (F,G) Clinical fundus photographs and scores of mice on Day 14 after immunization in the four groups mentioned above. White arrow, vasculitis. Red arrow, inflammatory exudation. (n = 5‐8/group; mean ± SD; NS p > 0.05, ^*^ *p* < 0.05; Kruskal–Wallis test, followed by pairwise Mann–Whitney U tests with Bonferroni correction). (H) Fundus fluorescein angiography with fluorescein sodium of mice on day 14 after immunization in the four groups mentioned above. White arrow, vessel leakage. Red arrow, vasculitis.

To assess the functional role of IFT122‐A773E in IPU, we generated knock‐in mice carrying the corresponding murine mutation (p.A825E; NM_031177.4). Sanger sequencing confirmed successful replacement of the endogenous allele with the mutant allele (Figure 2A). Long‐term monitoring showed no significant differences in body weight between mutant and WT mice (Figure S2A). Because ciliary gene mutations have been widely reported to cause renal abnormalities [28, 29, 30, 31, 32, 33, 34, 35]. we evaluated renal histology and function in both groups. We observed no significant differences in renal structure, serum urea nitrogen, creatinine, or uric acid levels (Figure S2B–E). Neither group exhibited inflammatory exudation (Figure S2F–H). These findings were consistent with the clinical presentation of the individual carrying the IFT122‐A773E mutation, who showed normal growth and renal function.

To determine whether IFT122‐A773E influences autoimmune uveitis severity, we induced EAU in mice through immunization with the interphotoreceptor retinoid‐binding protein. On day 14, mutant mice exhibited significantly higher clinical and histopathological scores than WT mice (Figure 2B–E). Histopathological examination revealed extensive inflammatory infiltration and retinal folding in mutant mice. Compared with WT mice, mutant mice exhibited increased inflammatory exudation and blurred retinal structures on fundus imaging, along with pronounced vascular tortuosity, thickening, and leakage on fluorescein angiography, indicating severe BRB disruption (Figure 2F–H).

Given that uveitis is an immune‐mediated disease, we next investigated whether the IFT122‐A773E mutation affects immune phenotypes. To this end, we collected spleens and cervical draining lymph nodes (CDLNs) from both WT mice and mutant mice for flow cytometric analysis. The results showed no difference concerning the frequency of splenic and CDLNs Th1 and Th17 cells between WT mice and mutant mice under both non‐immunized mice and EAU‐induced mice (Figure S2I–L). To further evaluate local immune responses, the retina was immunostained for CD4^+^ T cells and macrophages (F4/80^+^ or IBA1^+^). The results did not show any difference among CD4+ T cells, F4/80+ cells, and IBA1+ cells in the retina, nor between WT mice and mutant mice under either non‐immunized or EAU‐induced conditions (Figure 2M).

These results suggest that the aggravation EAU observed in IFT122‐A773E mutant mice is more likely driven by local retinal RPE cells, rather than by alternated immune phenotyping.

### 2.3 IFT122 A773E Impairs RPE Proliferation and Antioxidant Defense and Promotes Inflammatory Cytokine Secretion

**FIGURE 3:**
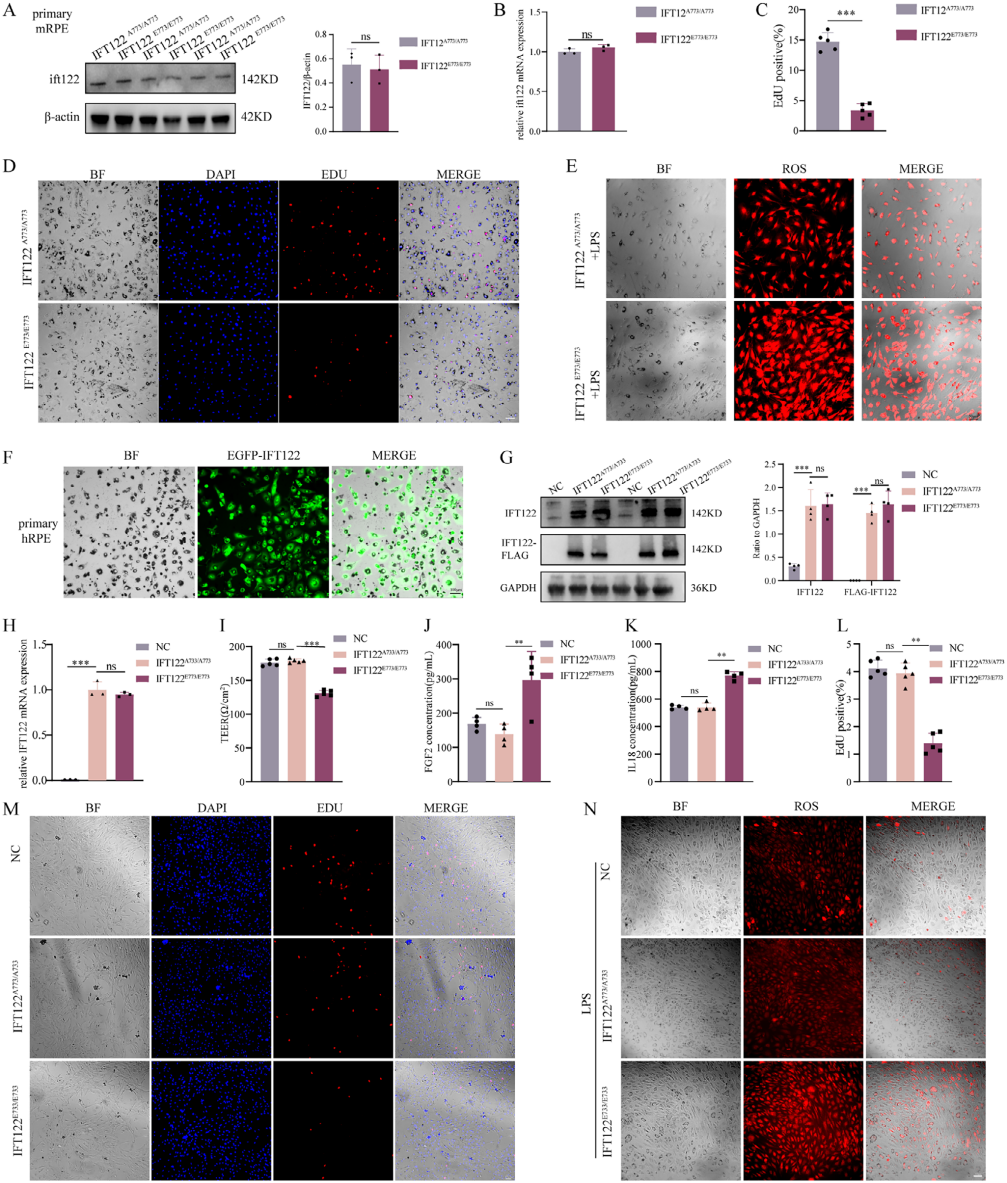
The effects of IFT122 mutation in primary RPE cells. (A,B) The protein and mRNA expression and quantification of ift122 in primary mice RPE (mRPE) cells isolated from wild‐type and homozygotes mice. (n = 3/group; mean ± SD; ^*^ *p* < 0.05, ^**^ *p* < 0.01; Unpaired Student's *t*‐test). (C,D) EdU positive immunofluorescence results and percentage in primary mRPE cells of wild type and homozygotes. (scale bar, 100 µm). (E) ROS‐positive cells in primary RPE cells of wild type and homozygotes. (scale bar, 50 µm). (F) Transfection efficiency of the IFT122 overexpression adenovirus in human primary RPE cells (BF, bright field; EGFP, enhanced green fluorescence protein; bar: 100 µm). (G,H) The protein and mRNA expression and quantification of IFT122 ^A773/A773^ and IFT122^E773/E773^ overexpression efficiency. (n = 3‐4/group; mean ± SD; NS > 0.05, ^*^ *p* < 0.05, ^**^ *p* < 0.01; one‐way ANOVA and Bonferroni post hoc test). (I) Trans‐epithelial electrical resistance (TEER) of primary human RPE (hRPE) cells with IFT122 ^A773/A773^ or IFT122^E773/E773^ overexpression and control cells. (n = 5/group; mean ± SD; NS > 0.05, ^*^ *p* < 0.05, ^**^ *p* < 0.01; one‐way ANOVA and Bonferroni post hoc test). (J) FGF2 concentration of hRPE in negative control(NC), IFT122 ^A773/A773^ and IFT122^E773/E773^ overexpression by ELISA. (n = 4–5/group; mean ± SD; NS > 0.05, ^*^ *p* < 0.05, ^**^ *p* < 0.01; one‐way ANOVA and Bonferroni post hoc test)). (K) IL18 concentration of hRPE by ELISA in the three groups mentioned above (n = 4–5/group; mean ± SD; NS > 0.05,^*^ *p* <0.05, ^**^ *p* < 0.01; one‐way ANOVA and Bonferroni post hoc test)). (L, M) EdU positive immunofluorescence results and percentage of hRPE in the three groups mentioned above(scale bar, 100 µm, n = 5‐6/group; mean ± SD; NS > 0.05, ^*^ *p* < 0.05, ^**^ *p* < 0.01; one‐way ANOVA and Bonferroni post hoc test)). (N) ROS‐positive cells in primary hRPE cells under LPS stimulation of the three groups mentioned above (scale bar, 100 µm).

To investigate the mechanism underlying the aggravated EAU phenotype, we isolated primary RPE cells from mutant and WT mice (Figure S3A). We found that IFT122 expression at both the mRNA and protein levels was comparable between groups. In addition, we examined IFT122 expression in isolated mouse RPE before and after EAU induction. Results showed IFT122 expression in primary RPE remained unchanged following EAU induction (Figure S3C). Consistent with observations in peripheral blood mononuclear cells from individuals carrying this mutation, neither the variant nor ocular inflammation alters IFT122 expression (Figure 3A,B).

IFT122 is a core component of the intraflagellar transport complex and participates in cellular processes, including cell‐cycle regulation, signal transduction, apoptosis, and transcriptional regulation [36, 37, 38]. We therefore assessed proliferation and antioxidant stress responses in RPE cells. Following lipopolysaccharide stimulation, mutant RPE cells exhibited increased ROS accumulation and reduced proliferative capacity compared with WT cells (Figure 3C–E). These findings indicated that the A773E variant impairs antioxidant defense and proliferative capacity.

To evaluate the effects in human cells, we cultured primary human RPE cells (Figure S3B). Next, we transduced RPE65‐positive cells with adenoviruses expressing enhanced green fluorescent protein‐tagged IFT122‐A773 or IFT122‐E773. Successful overexpression was confirmed using RT‐qPCR and western blotting (Figure 3F–H). Compared with controls, cells expressing IFT122‐E773 exhibited reduced transepithelial electrical resistance (TEER) and increased IL‐18 and fibroblast growth factor 2 (FGF2) secretion, as measured using enzyme‐linked immunosorbent assay (ELISA) (Figure 3I–K). Under lipopolysaccharide stimulation, IFT122‐E773‐expressing cells exhibited reduced proliferation and increased ROS accumulation (Figure 3L–N).

Collectively, the IFT122‐A773E variant impairs RPE proliferation, antioxidant defense, and barrier function while promoting proinflammatory cytokine secretion, thereby contributing to retinal inflammation and BRB disruption.

### 2.4 IFT122‐A773E Promotes Inflammatory Responses and Compromises Barrier Integrity in ARPE19 Cells

**FIGURE 4:**
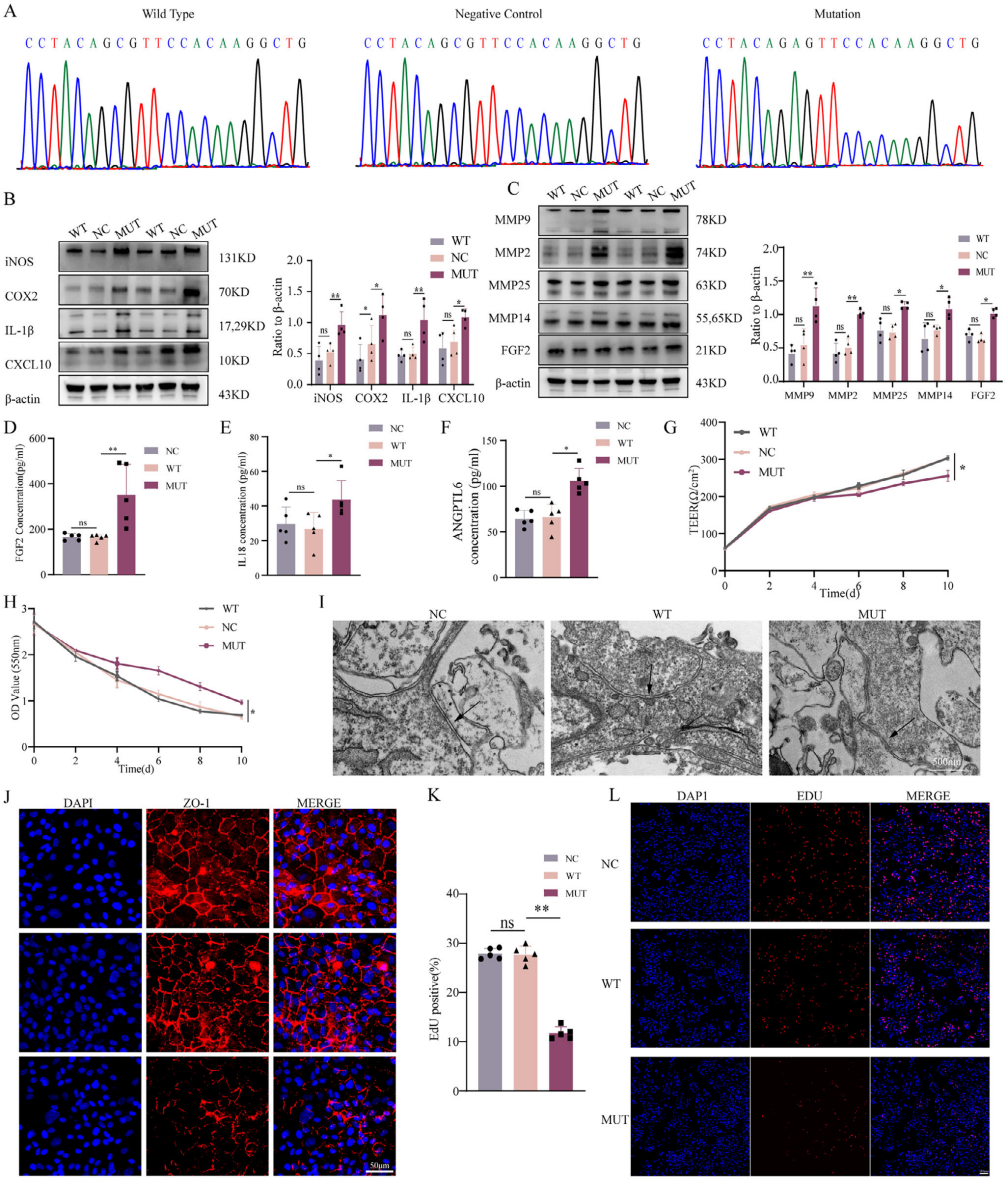
The effects of IFT122 mutation in ARPE‐19 cells. (A) Sanger sequencing of the IFT122‐A773E mutation in the ARPE19 cells, including wild type (WT), negative control (NC), and mutation (MUT). (B) Protein expression and quantification of iNOS, COX2, IL‐1β, and CXCL10 in WT, NC, and MUT cells. (n = 4/group; mean ± SD; NS p > 0.05, ^*^ *p* < 0.05, ^**^ *p* < 0.01; one‐way ANOVA and Bonferroni post hoc test). (C) Protein expression and quantification of MMP9, MMP2, MMP25, MMP14 and FGF2 in WT, NC and MUT cells. (n = 4/group; mean ± SD; NS p > 0.05, ^*^ *p* < 0.05, ^**^ *p* < 0.01; one‐way ANOVA and Bonferroni post hoc test). (D) FGF2 concentration of WT, NC and MUT cells. (n = 5/group; mean ± SD; NS p > 0.05, ^*^ *p* < 0.05, ^**^ *p* < 0.01; one‐way ANOVA and Bonferroni post hoc test). (E) IL18 concentration of WT, NC, and MUT cells. (n = 5/group; mean ± SD; NS *p* > 0.05, ^*^ *p* < 0.05, ^**^ *p* < 0.01; one‐way ANOVA and Bonferroni post hoc test). (F) ANGPTL6 concentration of WT, NC, and MUT cells. (n = 5/group; mean ± SD; NS p > 0.05, ^*^ *p* < 0.05, ^**^ *p* < 0.01; one‐way ANOVA and Bonferroni post hoc test). (G) TEER of WT, NC and MUT cells. (n = 5‐8/group; mean ± SD; NS > 0.05, ^*^ *p* < 0.05, ^**^ *p* < 0.01; one‐way ANOVA and Bonferroni post hoc test)). (H) The absorbance at 550 nm of the three groups mentioned above in the phenol red leakage assay. (n = 5–8/group; mean ± SD; NS> 0.05, ^*^ *p* < 0.05, ^**^ *p* < 0.01; one‐way ANOVA and Bonferroni post hoc test)). (I) Images of transmission electron microscope (TEM) in the three groups mentioned above. Black arrow, membrane fusion points. (scale bar, 500 nm). (J) The immunofluorescence results of ZO‐1 in the three groups mentioned above (scale bar, 50 µm). (K,L) EdU positive immunofluorescence results in the three groups mentioned above.

To assess the effect of the IFT122‐A773E variant on RPE cells, we established a CRISPR‐Cas9‐engineered ARPE19 cell line carrying the heterozygous mutation (MUT), a parallel isogenic line generated using the same editing procedure but lacking the mutation (negative control [NC]), and WT ARPE19 cells. Sanger sequencing confirmed successful introduction of the mutation (Figure 4A). Morphological assessment using an inverted microscope revealed structural alterations in MUT cells compared with NC and WT cells, indicating mutation‐associated phenotypic alterations (Figure S4A). Although IFT122 facilitates ciliogenesis [36, 37], we observed no obvious differences in cilia formation among the three groups upon confocal microscopy (Figure S4B).

Protein analyses demonstrated that the IFT122‐A773E variant enhanced inflammatory and matrix‐remodeling responses. Proinflammatory mediators, including IL‐1β, inducible nitric oxide synthase, cyclooxygenase‐2, and C‐X‐C motif chemokine ligand 10, were significantly upregulated in MUT cells compared with those in NC and WT cells (Figure 4B). Matrix metalloproteinases (MMPs; MMP2, MMP9, MMP14, and MMP25) and the angiogenesis‐related factor FGF2 were also significantly upregulated, reflecting increased extracellular matrix degradation and angiogenic potential (Figure 4C). Consistently, ELISA demonstrated increased secretion of IL‐18, FGF2, and angiopoietin‐like 6 (ANGPTL6) in MUT cells (Figure 4D–F). These findings indicated that the IFT122‐A773E variant promotes a proinflammatory and proangiogenic microenvironment that facilitates extracellular matrix remodeling, potentially contributing to BRB disruption and retinal neovascularization.

To determine whether the IFT122‐A773E variant affects epithelial barrier integrity, we performed TEER measurements and observed a significant reduction in MUT cells compared with that in NC and WT cells. Phenolsulfonphthalein leakage assays further demonstrated increased permeability in MUT cells (Figure 4G,H). Immunofluorescence analysis revealed significant downregulation of zonula occludens‐1 in MUT cells, and transmission electron microscopy showed blurred membrane fusion points and thickened, discontinuous tight junctions (Figure 4I,J). Additionally, MUT cells exhibited reduced proliferative capacity compared with NC and WT cells (Figure 4K,L).

Thus, the IFT122‐A773E variant in RPE cells enhances inflammatory responses and matrix remodeling while impairing cell proliferation and barrier integrity, thereby contributing to BRB breakdown, severe retinal inflammation, and increased susceptibility to IPU.

### 2.5 IFT122‐A773E Facilitates Inflammatory Responses Via the MEK/ERK/FRA1 Pathway

**FIGURE 5:**
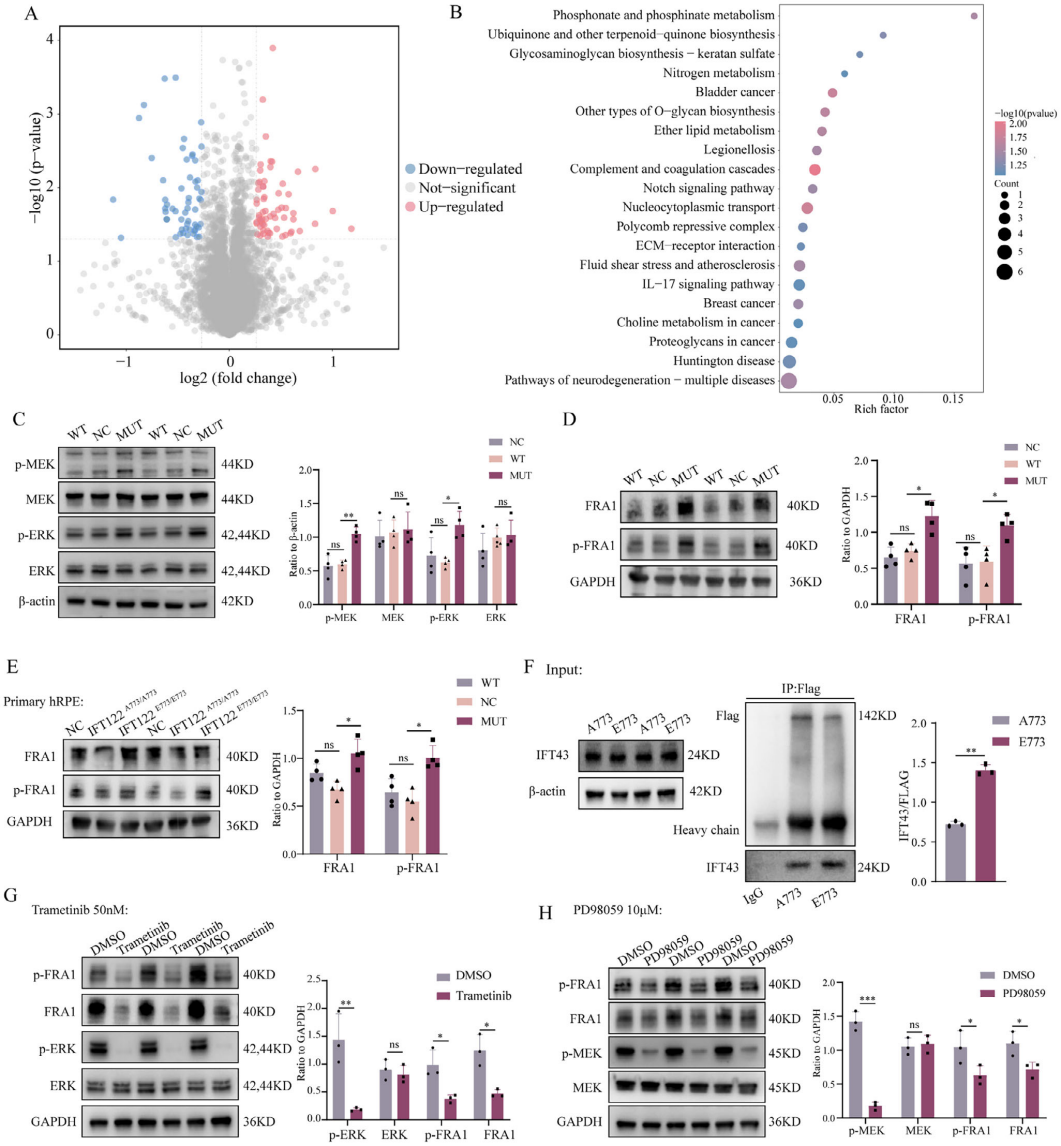
Activation of MEK/ERK pathway after IFT122‐A773E mutation. (A) The volcanic map of differentially expressed genes (DEGs) between WT and MUT ARPE19 cells. (B) The KEGG enrichment of DEGs between WT and MUT ARPE19 cells. (C) Protein levels and quantitative charts of p‐MEK, MEK, p‐ERK, and ERK in WT, NC, and MUT cells (n = 4/group; mean ± SD; ^**^ *p* < 0.01; one‐way ANOVA and Bonferroni post hoc test). (D) Protein levels and quantitative charts of FRA1, p‐FRA1 in WT, NC, and MUT cells (n = 4/group; mean ± SD; ^**^ *p* < 0.01; one‐way ANOVA, Bonferroni post hoc test). (E) Protein levels and quantitative charts of FRA1, p‐FRA1 of primary human RPE (hRPE) cells with IFT122 ^A773/A773^, IFT122^E773/E773^ overexpression and control cells. (n = 4/group; mean ± SD; ^**^ *p* < 0.01; one‐way ANOVA and Bonferroni post hoc test). (F) Co‐IP results of IFT122 to IFT43. (n = 3/group; mean ± SD; ^**^ *p* < 0.01; unpaired Student's *t*‐test). (G) Protein levels and quantitative charts of FRA1, p‐FRA1, p‐ERK and ERK after Trametinib treatment. (n = 3/group; mean ± SD; ^**^ *p* < 0.01; unpaired Student's t‐test). (H) Protein levels and quantitative charts of FRA1, p‐FRA1, p‐MEK and MEK after Trametinib treatment. (n = 3/group; mean ± SD; ^**^ *p* < 0.01; unpaired Student's *t*‐test).

To explore the molecular mechanisms underlying the EAU phenotype, we performed data‐independent acquisition proteomic profiling of MUT and WT ARPE‐19 cells. We detected a total of 8537 proteins, among which 120 were differentially expressed, including 62 upregulated and 58 downregulated proteins in MUT cells compared with those in WT cells (Figure 5A,B). Kyoto Encyclopedia of Genes and Genomes (KEGG) pathway enrichment analysis revealed significant enrichment of phosphorylation‐dependent signaling cascades. Gene Ontology (GO) enrichment analysis showed that differentially expressed proteins were primarily associated with metabolic pathways and intracellular organelles (Figure S5A). Ciliary proteins are recognized regulators of phosphorylation‐mediated signaling pathways, including MEK, ERK, phosphoinositide 3‐kinase, and rat sarcoma (RAS) [38, 39, 40]. We therefore investigated signaling networks interacting with the IFT protein family, including MEK, signal transducer and activator of transcription 3, RAS, protein kinase C, and FBJ murine osteosarcoma viral oncogene homolog (FOS). Phosphorylated MEK and ERK levels were significantly increased in MUT cells (Figure 5C, Figure S5B,C). Within the IL‐17 signaling pathway, FRA1 protein levels were significantly elevated in MUT cells, as confirmed using western blotting. Phosphorylated FRA1 (p‐FRA1) levels were also significantly increased in both MUT ARPE‐19 and primary human RPE cells (Figure 5D,E).

Because IFT122 links the core and peripheral subcomplexes to form the complete IFT‐A complex, we performed coimmunoprecipitation to evaluate the interaction between IFT122 and its canonical partner IFT43 [41, 42]. Coimmunoprecipitation of FLAG‐tagged IFT122‐A773 and IFT122‐E773 confirmed the interaction of IFT122 with IFT43. Notably, the IFT122‐A773E variant exhibited stronger binding to IFT43 than IFT122‐A773, indicating that the mutation enhances IFT‐A complex stability and may alter ciliary signaling dynamics (Figure 5F). We next sought to identify the molecular bridges between the IFT122–IFT43 binding and MEK/ERK activation. We reanalyzed the proteomic results and identified a dysregulated calcium signaling pathway in MUT groups (Figure S5D). Therefore, intracellular calcium levels in WT, NC, and MUT cells were measured using Fluo‐4 AM staining. The results showed a higher intracellular calcium level in MUT cells (Figure S5E). To evaluate the effect of intracellular calcium on MEK/ERK pathway, MUT cells were treated with the calcium chelator BAPTA‐AM. The results showed that BAPTA‐AM significantly reduced MEK and ERK phosphorylation levels (Figure S5F–H).

As calcium signaling appeared to contribute to MEK/ERK activation, we next investigated downstream genes regulated by this pathway. MEK activation promotes ERK phosphorylation and activates transcription factors such as activator protein‐1 (AP‐1) and nuclear factor kappa B (NF‐κB) [43, 44, 45]. FRA1 is a key component of the AP‐1 complex and regulates cell proliferation, differentiation, and transdifferentiation [46, 47, 48]. Treatment of MUT ARPE‐19 cells with selective MEK/ERK inhibitors (trametinib and PD98059) significantly reduced total FRA1 and p‐FRA1 levels, confirming that MEK/ERK signaling drives FRA1 activation (Figure 5G,H).

Collectively, these results reveal that enhanced IFT122–IFT43 binding caused by the A773 mutation may alter IFT‐A complex function, consequently leading to an increased intracellular calcium level and in turn to activation of MEK/ERK/FRA1 inflammation axis.

### 2.6 FRA1 Regulates Inflammation and Matrix Degradation Capacity via Transcriptional Control

**FIGURE 6:**
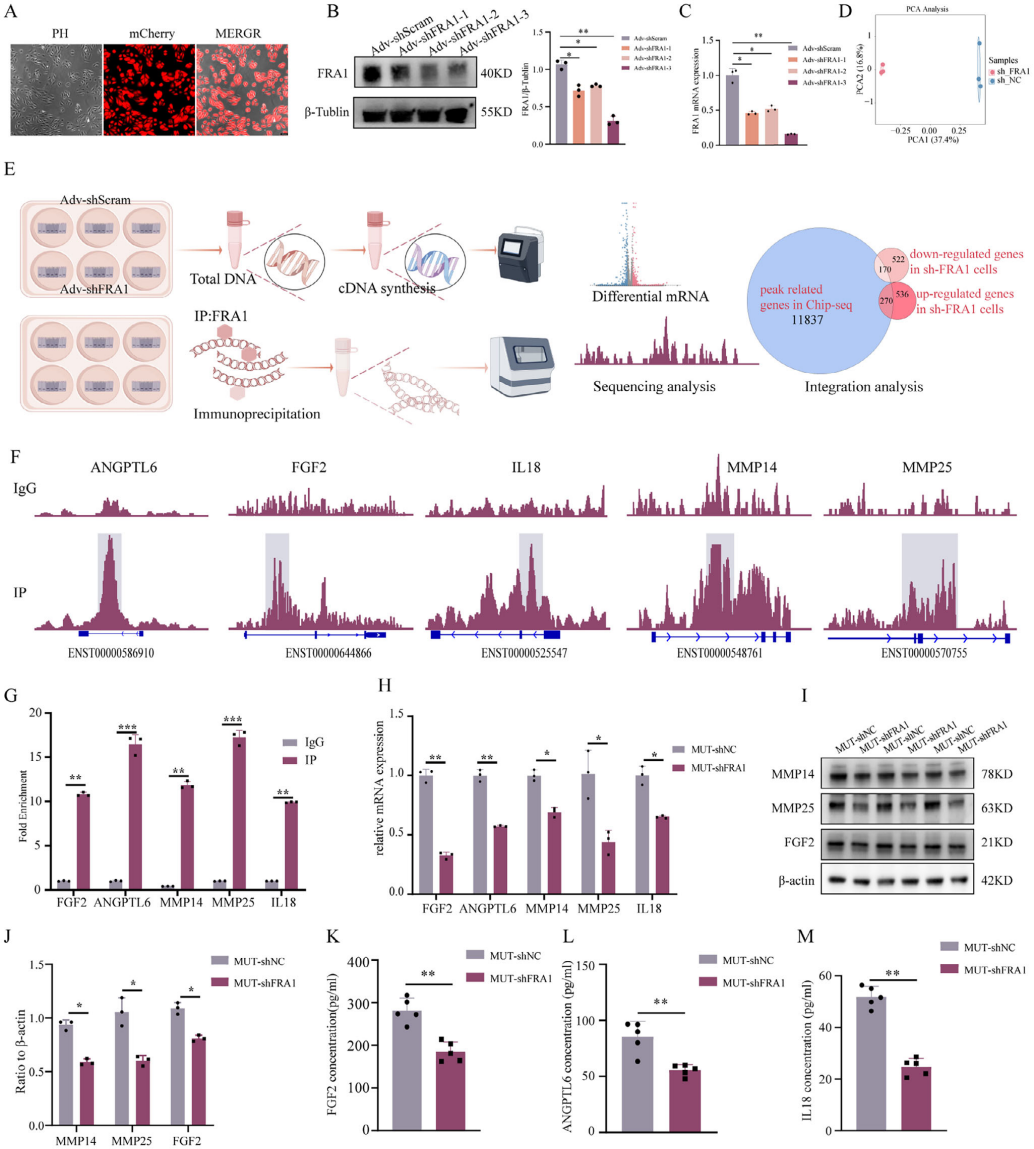
Alleviated EAU inflammation after FRA1 silencing. (A–C) Transfection efficiency in protein and mRNA levels of the FRA1 knockdown adenovirus in RPE cells (n = 3‐4/group; mean ± SD; ^**^ *p* < 0.01; one‐way ANOVA, Bonferroni post hoc test; bar: 100 µm). (D) PCA between Scram cells (ShNC) and FRA1 knockdown (sh‐FRA1) cells. (E) Integrated analysis of Chip‐seq and RNA‐seq Data. (F) The peak plots ANGPTL6, FGF2, IL18, MMP14, and MMP25 according to the ChIP‐seq. (G) CHIP‐qPCR results of the five mediators mentioned above. (n = 3/group; mean ± SD; ^**^ *p* < 0.01; Student's *t*‐test). (H) qPCR results of the five mediators mentioned above in the shNC and sh‐FRA1 MUT cells. (n = 3/group; mean ± SD; ^**^ *p* < 0.01; Unpaired Student’ s *t*‐test). (I,J) Protein levels and quantitative charts of MMP14, MMP25, and FGF2 of the shNC and sh‐FRA1 MUT cells. (n = 3/group; mean ± SD; ^**^ *p* < 0.01; Unpaired Student's *t*‐test). (K‐M) FGF2, IL18 and ANGPTL6 concentration of the shNC and sh‐FRA1 MUT cells. (n = 4‐6/group; mean ± SD; ^**^ *p* < 0.01; Unpaired Student’ s *t*‐test).

To investigate downstream mechanisms of the MEK/ERK/FRA1 pathway, we constructed an adenoviral vector carrying FRA1‐specific short hairpin RNA (Adv‐shFRA1‐1, Adv‐shFRA1‐2, and Adv‐shFRA1‐3) and evaluated knockdown efficiency at both the mRNA and protein levels. Adv‐shFRA1‐3 achieved the highest silencing efficiency (Figure 6A–C). RNA sequencing (RNA‐seq) revealed clear separation between control and FRA1‐knockdown groups in principal component analysis, indicating substantial transcriptional reprogramming (Figure 6D). A total of 925 genes were significantly dysregulated, including 499 upregulated and 426 downregulated transcripts. KEGG enrichment analysis showed that differentially expressed genes were primarily associated with nucleotide binding, transferase activity, DNA binding, microtubule binding, RNA binding, and chromatin binding (Figure S6A,B). GO enrichment analysis further identified biological processes related to cell cycle progression, cell division, transcriptional regulation, chromosome segregation, DNA damage response, and RNA polymerase II‐mediated transcription (Figure S6C). These results highlight the role of FRA1 as a transcription factor that regulates gene expression through promoter binding.

To identify direct genomic targets of FRA1, we performed chromatin immunoprecipitation sequencing (ChIP‐seq) (Figure 6E). ChIP‐seq analysis confirmed that FRA1 binds to transcription start sites of genes involved in inflammation, extracellular matrix remodeling, and angiogenesis. Specifically, FRA1 directly targeted promoters of inflammatory cytokines (IL‐17 and IL‐18), chemokines (CXCL3, CXCL8, and CXCL16), fibroblast growth factors (FGF2 and FGF5), and vascular endothelial growth factor family members. Integrative analysis of ChIP‐seq and RNA‐seq datasets revealed that FRA1 knockdown significantly reduced the mRNA levels of FGF2, IL‐18, MMP‐14, MMP‐25, and ANGPTL6 (Figure 6F). ChIP‐qPCR confirmed direct binding of FRA1 to the transcription start sites of these genes (Figure 6G). Western blotting and ELISA further demonstrated reduced protein expression following FRA1 knockdown (Figure 6H–M).

Collectively, these findings indicate that FRA1 functions as a central transcriptional effector downstream of MEK/ERK signaling, regulating inflammatory mediators, matrix metalloproteinases, and angiogenic factors in RPE cells, thereby identifying FRA1 as a potential therapeutic target in IPU.

### 2.7 FRA1 Inhibition in IFT122 Knock‐in Mice Attenuates EAU Inflammation

**FIGURE 7:**
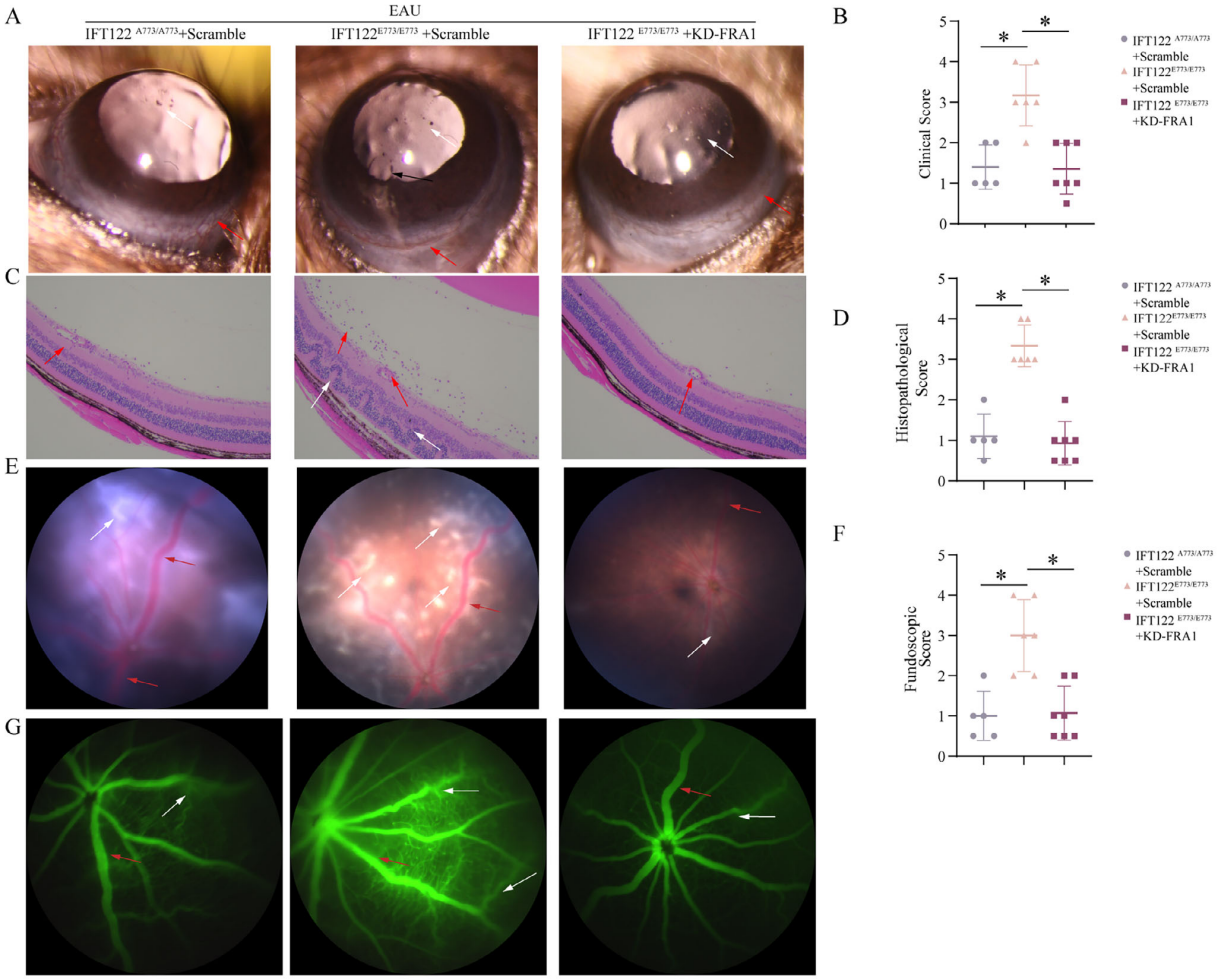
Alleviated EAU inflammatory phenotypes after FAR1 silencing. (A,B) Anterior segment inflammation and clinical scores of mice in wild type treated with scramble AAV, IFT122^E773/E773^ with scramble AAV and IFT122^E773/E773^ treated with FRA1 knock‐down AAV. White arrow, corneal opacity, exudate. Red arrow, ciliary hyperemia. Black arrow, irregular pupils and posterior synechiae (n = 5–8/group; mean ± SD; NS *p* > 0.05; Kruskal–Wallis test, followed by pairwise Mann–Whitney U tests with Bonferroni correction). (C,D) Hematoxylin‐eosin staining and pathological scores of mice in the three groups mentioned above. White arrow, retina fold; Red arrow, inflammatory cells and exudate. (n = 5‐8/group; mean ± SD; NS p > 0.05, ^*^ *p* < 0.05; Kruskal–Wallis test, followed by pairwise Mann–Whitney U tests with Bonferroni correction). (E,F) Clinical fundus photographs and scores of mice on day 14 after immunization in the three groups mentioned above. White arrow, vasculitis. Red arrow, inflammatory exudation. (n = 5‐8/group; mean ± SD; NS *p* > 0.05, ^*^ *p* < 0.05; Kruskal–Wallis test, followed by pairwise Mann–Whitney U tests with Bonferroni correction). (G) Fundus fluorescein angiography with fluorescein Sodium of mice on day 14 after immunization in the three groups mentioned above. White arrow, vessel leakage. Red arrow, vasculitis.

Given the central role of FRA1 in regulating inflammation and matrix remodeling, we examined whether FRA1 suppression alleviates EAU pathology in IFT122‐A773E knock‐in mice. A mouse‐specific adeno‐associated virus (AAV) targeting FRA1 was delivered via subretinal injection (Figure S7A). AAV‐mediated FRA1 knockdown reached peak efficiency at 3–4 weeks after injection and remained stable for > 2 months (Figure S7B–D). Following EAU induction, FRA1‐silenced mice exhibited significantly reduced clinical and histopathological inflammation compared with that in vector‐treated controls (Figure 7A–D). Specifically, FRA1‐silenced mice showed reduced anterior segment inflammation and hyperemia, along with fewer inflammatory cells and exudates on histological examination. Fundus imaging demonstrated significantly reduced vascular leakage and vasculitis in the FRA1‐silenced group (Figure 7E,F).

**FIGURE 8:**
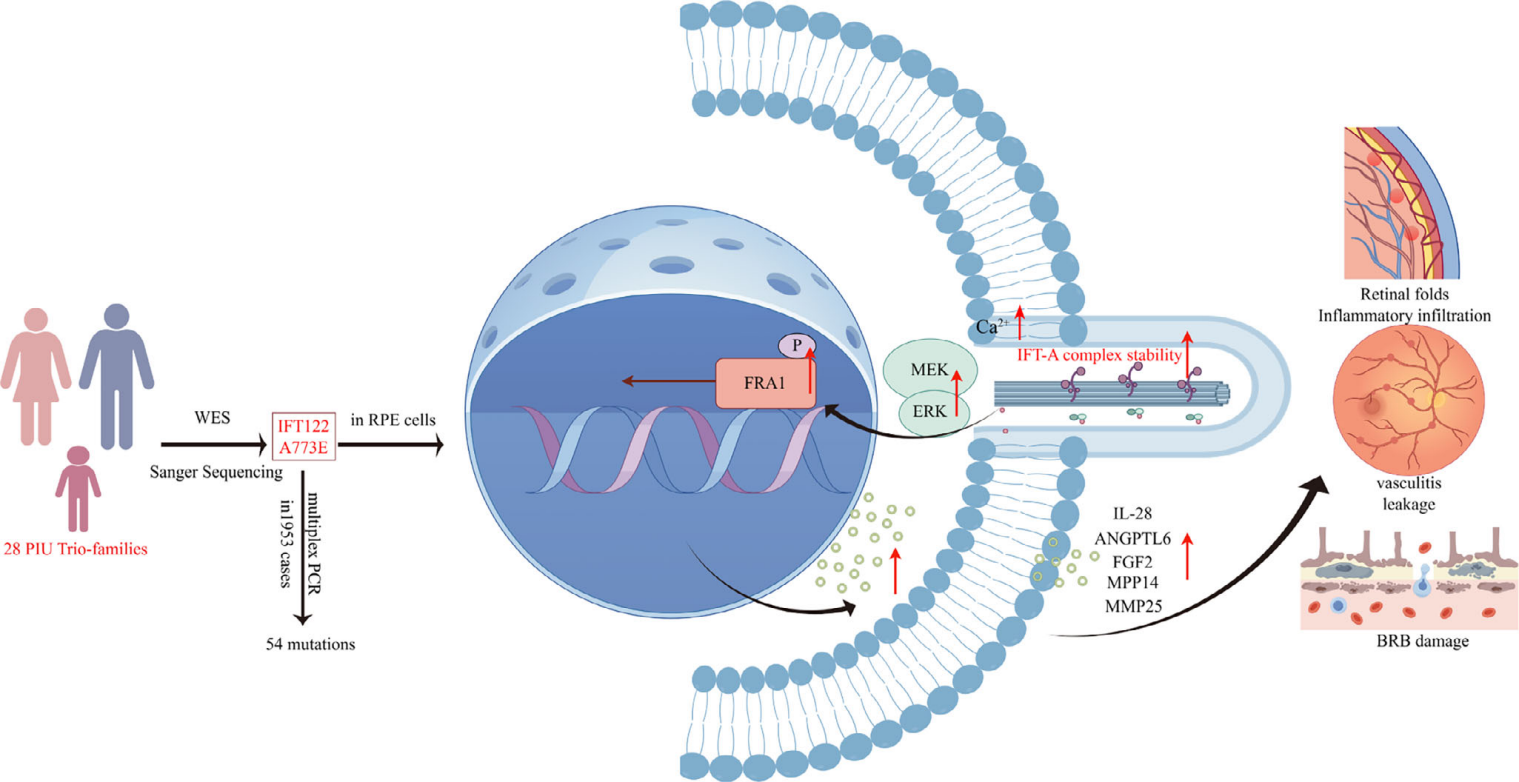
The schematic diagram illustrates the molecular mechanism of how the IFT122‐A773E variant contributed to inflammation and BRB damage in IPU.

Thus, AAV‐mediated FRA1 inhibition significantly attenuated the inflammatory phenotype in IFT122‐A773E knock‐in mice, highlighting the MEK/ERK/FRA1 axis as a potential therapeutic target for IPU (Figure 8A).

## 3 Discussion

Genetic susceptibility is increasingly recognized as an important contributor to autoimmune diseases, while the genetic basis and underlying mechanisms of IPU remain poorly understood. In this study, we identified a rare de novo IFT122‐A773E variant through trio‐based WES and demonstrated its pathogenic relevance using in vivo and in vitro models. Mechanistically, our data suggest that the A773E variant impairs ciliary signaling, resulting in intracellular calcium accumulation and subsequent activation of the MEK/ERK pathway, which in turn enhances FRA1 transcriptional activity. Collectively, these findings identify IFT122‐A773E as a potential pathogenic variant in IPU and reveal a previously unrecognized molecular pathway that contributes to disease progression.

To address the role of genetic susceptibility in IPU, we conducted trio‐based WES in 28 trios with IPU and multiplex PCR‐based sequencing in sporadic cases from a Han Chinese cohort. We identified 33 de novo mutations, among which IFT122‐A773E was the rarest and the most potentially pathogenic. Although additional IFT122 variants were detected, computational pathogenicity analyses suggested that most of these variants are likely benign or of uncertain significance. Therefore, in the present study, we focused on the exceptionally rare de novo A773E variant, which showed consistent genetic, structural, and functional evidence of potential pathogenicity.

IFT122 is an intraflagellar transport protein that plays an essential role in primary cilium assembly and cilia‐dependent signal transduction [49, 50]. It has been established that IFT122 dysfunction contributes to RPE degeneration and functional impairment, whereas complete loss of IFT122 disrupts ciliogenesis [42, 51, 52, 53]. Given that IPU is a typical immune‐mediated ocular disease, we first sought to determine whether the IFT122‐A773E variant contributes to disease pathogenesis by altering immune phenotyping. Surprisingly, neither the proportions of total CD4^+^ T, including Th1 and Th17, nor the infiltration of CD4^+^ T cells or macrophages in the retina was affected by this mutation. These findings suggest that the IFT122‐A773E variant does not primarily act through direct immune cell modulation, but instead through resident retinal cells, particularly RPE cells. Notably, unlike ciliogenesis damage in loss of IFT122, the A773E variant did not impair ciliogenesis in RPE in our study. Functional studies demonstrated that IFT122‐A773E impaired RPE proliferation, disrupted epithelial barrier integrity, and enhanced pro‐inflammatory responses, both in primary human RPE cells and CRISPR/Cas9‐engineered ARPE‐19 cells. Furthermore, in the EAU model, IFT122‐A773E knock‐in mice developed more severe ocular inflammation, characterized by increased inflammatory exudation, BRB leakage, and retinal structural disorganization. This cross‐model consistency substantially strengthens the conclusion that IFT122‐A773E contributes to inflammatory and barrier‐related abnormalities in RPE cells.

To further elucidate the molecular mechanisms underlying IFT122‐A773E‐mediated RPE inflammation, we performed proteomic sequencing and identified several dysregulated cilia‐associated signaling pathways. Among these, phosphorylation‐related pathways were the most significantly enriched. Our subsequent validation experiments confirmed the aberrant activation of the MEK/ERK signaling cascade. In addition, calcium signaling, a classical second messenger downstream of primary cilia, was also significantly enriched [54]. It has been shown that intracellular Ca^2^^+^ is an upstream trigger of MEK/ERK activation, and our findings also suggest that the intracellular calcium accumulation leads to abnormal activation of the MEK/ERK pathway [55]. Activated MEK/ERK signaling subsequently enhanced the transcriptional activity of FRA1. Notably, FRA1 was enriched in the IL‐17 signaling pathway, a central driver of autoimmune inflammation that has been strongly implicated in the pathogenesis of uveitis [10, 56]. Moreover, FRA1 is a well‐established pro‐inflammatory transcription factor that regulates a broad spectrum of inflammatory mediators involved in autoimmune tissue injury [46, 57]. In our study, mechanistic analyses with CHIP‐seq and RNA‐seq demonstrated that FRA1 directly regulates downstream targets, including FGF2, IL‐18, MMP‐14, MMP‐25, and ANGPTL6, thereby amplifying local retinal inflammatory responses and contributing to disease progression in RPE cells. In addition, FRA1 knockdown significantly attenuated the expression of these target genes in RPE cells, and alleviated pathological features in IFT122‐A773E knock‐in EAU model mice. Taken together, we propose a previously unrecognized pathogenic model in which the Ca^2^^+^/MEK/ERK/FRA1 signaling axis conducted by IFT122‐A773E connecting ciliary dysfunction with autoimmune ocular inflammation.

Despite these advances, several limitations of the present study warrant consideration. First, the current findings focused on a single de novo variant, and whether other *IFT122* variants identified in the sporadic cohort contribute to IPU pathogenesis remains unknown. Second, given that *IFT122* is broadly expressed across multiple human tissues, including the testes, bones, sensory organs, and connective tissues, the potential systemic consequences of this variant beyond the ocular compartment require further investigation. Third, although *IFT122* is highly conserved between humans and mice, species‐specific differences in expression patterns, cellular context, and downstream signaling may exist. Most critically, the precise molecular mechanism by which the A773E substitution elevates calcium levels remains incompletely elucidated. More studies are expected to address these issues.

In summary, we identified a rare de novo *IFT122* mutation (c. 2318 C>A; NM_052990) in an IPU trio, and validated its pathogenicity across multiple experimental models. Functional and mechanistic analyses revealed that this variant elevates intracellular calcium levels and aberrantly activates the MEK/ERK/FRA1 signaling axis, thereby transcriptionally upregulating a network of inflammatory, angiogenic, and matrix‐remodeling genes that collectively exacerbate disease progression. These findings establish a mechanistic association between ciliary dysfunction and autoimmune uveitis, highlight FRA1 as a previously unrecognized mediator of IFT122‐driven retinal inflammation, and provide novel genetic insights into IPU pathogenesis.

## 4 Materials and Methods

### 4.1 Ethics Statement and Patient Recruitment

This study has received approval from the Ethics Committee of the First Affiliated Hospital of Chongqing Medical University (Permit No.:201008, 2019‐100‐2, 2021–222) for all the human and mouse procedures. All research activities complied with the ethical guidelines of the Declaration of Helsinki, and informed consent in writing was secured from participants or their guardians before they were enrolled. A total of 28 trios (each including one child with IPU and two unaffected parents) and 1953 sporadic cases of IPU were recruited from the First Affiliated Hospital of Chongqing Medical University. IPU is defined as PU (onset before age 16) without a specific clinical classification. This study excluded children diagnosed with Behçet's disease, VKH disease, systemic underlying conditions, or other clearly defined types of uveitis (toxoplasmosis, tuberculosis, syphilis, etc.) according to the clinical manifestation and relevant laboratory examinations.

### 4.2 Whole‐Exome Sequencing (WES)

Genomic DNA was extracted from peripheral blood using the QIAGEN QIAamp DNA Mini Blood Kit and quantified with the Qubit dsDNA BR Assay Kit. DNA quality was confirmed by agarose gel electrophoresis. Sequencing libraries were prepared using standard protocols, and exome capture was performed with the NimbleGen SeqCap EX Exome Library v3.0 kit. Library quality and concentration were assessed before sequencing, and paired‐end 150 bp reads were generated on an Illumina HiSeq Xten platform. Short reads were aligned to the human genome (UCSC hg19) with Burrows‐Wheeler Aligner v0.7.15. The calling of variants was accomplished by GATKv3.6 and filtered by Hard filtering and Variant Quality Score Recalibration, and further annotated using ANNOVAR. All nonsynonymous variants within the coding and splicing regions were screened with dbSNP, 1000 Genomes Project (1000G), and NHLBI Exome Sequencing Project (ESP6500). Then, potential functional consequences were evaluated by SIFT, Mutationtaster, PolyPhen‐2, CADD, MCAP, and gerp++gt2 score.

### 4.3 Sanger Sequencing

Candidate de novo variants identified by whole‐exome sequencing in the affected child and unaffected parents underwent Sanger sequencing validation. Independent primer designed to amplify the target region, then amplification products were sequenced by Sangon Biotech (Shanghai, China) on Applied Biosystems 3730XL instrumentation (Thermo Fisher Scientific, USA) following standard operating procedures. The resulting sequencing was aligned and assessed by ClustalX software.

### 4.4 Multiplex PCR and Sequencing

Genomic DNA was extracted using the Rapid Blood Genomic DNA Isolation Kit (Sangon, Shanghai, China) according to the manufacturer's protocol. DNA concentration was quantified using a Qubit 2.0 Fluorometer (Thermo Fisher Scientific, USA) to ensure sufficient yields of high‐quality genomic DNA. A customized panel comprising 48 target single nucleotide polymorphism (SNP) sites was designed. Library preparation was carried out using a two‐step polymerase chain reaction (PCR) protocol, and the resulting amplicon products were purified using AMPure XP beads (Beckman Coulter, USA). The purified libraries were quantified and subsequently pooled. Paired‐end sequencing was conducted on a HiSeq X Ten platform (Illumina, San Diego, CA, USA). Raw sequencing reads were processed in two steps: (1) adapter sequences were trimmed using Cutadapt (v1.2.1), and (2) low‐quality bases (Q < 20) were removed from the 3′ to 5′ ends using PRINSEQ‐lite (v0.20.3). The resulting high‐quality reads were aligned to the human reference genome using BWA (v0.7.13‐r1126) with default parameters. SAMtools (v0.1.18) was used to determine the genotypes of the target sites, and ANNOVAR (2018‐04‐16) was employed for variant annotation and detection.

Genomic DNA was isolated from peripheral blood using a commercial kit and quantified to ensure sufficient quality. A custom panel targeting 48 SNP sites was designed, and libraries were prepared using a two‐step PCR approach. After purification and quantification, libraries were pooled and subjected to paired‐end sequencing on an Illumina HiSeq X Ten platform. Sequencing reads were trimmed, filtered for quality, and aligned to the human reference genome. Variants at the target sites were identified and annotated using standard bioinformatics tools with the help of Sangon Biotech (Shanghai)Co., Ltd.

### 4.5 Experimental Autoimmune Uveitis (EAU) Induction and Assessment

Female C57BL/6J mice were immunized with 350 µg human IRBP651–670 peptide (LAQGAYRTAVDLESLASQLT; Sangon Biotech, Shanghai) via subcutaneous injection. The peptide was dissolved in phosphate‐buffered saline and combined with equal‐volume complete Freund's adjuvant (CFA; Sigma–Aldrich, USA) containing Mycobacterium tuberculosis H37Ra (BD Biosciences). Concurrently, each mouse received 1 µg Bordetella pertussis toxin (Sigma‐Aldrich, USA) dissolved in 200 µL saline solution intraperitoneally. On day 14 following immunization, EAU clinical manifestations were evaluated via slit‐lamp examination and fundoscopy, which were scored independently by two blinded observers according to Caspi's 0.5‐point increment scale [58].

### 4.6 Primary Retinal Pigment Epithelium (RPE) Collecting and Culturing

Primary RPE cells were immediately obtained from 4‐week‐old C57BL/6J mice following euthanasia. Eyes were carefully enucleated and transferred to ice‐cold PBS. Under a stereomicroscope, the anterior segment, neural retina, and other connective tissues were removed clearly, leaving the posterior eyecup. The eyecups were then incubated in trypsin solution at 37°C for 35 min, and the pigmented RPE layer was gently detached by softly tapping with a pipette. The harvested cells were cultured in DMEM/F12 supplemented with 20% FBS and 1% penicillin–streptomycin. Human primary RPE cells were obtained through an identical procedure, except that trypsin digestion was shortened to 25 min. Primary RPE cells displayed a flat, polygonal shape, with cytoplasm containing abundant brown‐black pigment granules. Cells reached full confluence within 1–2 weeks. After four passages, the cytochrome content in primary cells gradually decreased. All primary cells used in the experiment were confirmed to remain viable within the four passage.

### 4.7 Cell Culture

ARPE19 cells were obtained from the American Type Culture Collection (ATCC, USA) and maintained in DMEM/F12 medium supplemented with 10% fetal bovine serum (FBS) at 37°C in a humidified incubator containing 5% CO_2_. Primary RPE cells were initially cultured in DMEM/F12 medium supplemented with 20% FBS and 1% penicillin–streptomycin. Once the first passage reached stability and could be subcultured, the culture medium was adjusted to contain 10% FBS for subsequent passages.

### 4.8 Adenovirus‐Mediated Cell Transduction

Adenoviral vectors targeting IFT122 and FRA1 were utilized to induce overexpression or knockdown of the respective genes, while corresponding control vectors served as negative controls. Expression and packaging vectors were transduced into ARPE19 cells at a multiplicity of infection of 25–35, following the manufacturer's instructions. Successful transduction was confirmed by fluorescence imaging and subsequent expression analysis.

### 4.9 CRISPR/Cas9 Gene Editing Technology

The Ift122 p.A825E point mutation mice were generated by Cyagen Company (Suzhou, China). Cas9 protein, one gRNA (gRNA‐A1: ATGGCCCTTAGCCTTCCACAAGG), and a donor oligo containing p.A825E (GCC to GAG) were co‐injected into fertilized eggs. The embryos were transferred to recipient female mice to obtain F0 mice. The genotype of point mutation mice was confirmed by PCR using a pair of primers (F1: 5’‐TTATTTATAGCCTGCGGCCTGAGA‐3’, R1:’‐GACAAAACACTCAATAAAC AAGCCC‐3’) and sequencing. F0 founder mice were crossed to C57BL/6J mice to produce heterozygous F1.

The ARPE‐19 cell line with the human IFT122 (p.A773E) gene point mutation was generated by Cyagen Company (Suzhou, China). The nuclease and guide molecule (CCAGCCTTGTGGAACGCTGTAGG) were incubated together to form an RNP complex. The donor oligo containing the p.A773E (GCG to GAG) mutation was transfected into the target cells. Monoclonal cells were prepared by limiting dilution, and the well‐conditioned monoclonal cells were identified by PCR and sequencing (F: TTTCACTCTTAGACTTTGCCCTGG, R: GATGATACACCAGATTACGCCACT), and positive clones were obtained.

### 4.10 Hematoxylin and Eosin (H and E) Staining

Fourteen days post‐immunization, euthanized mice underwent bilateral ocular enucleation. The collected ocular tissues were fixed by immersion in phosphate‐buffered saline (PBS) containing 10% formaldehyde and 5% glacial acetic acid. Following dehydration, tissues were embedded in paraffin and sectioned at 4–6 µm thickness. These sections underwent hematoxylin and eosin (H&E) staining, and histopathological assessment was performed according to Caspi's established criteria.

### 4.11 Fluorescein Fundus Angiography

Mice received an intraperitoneal injection of 1% sodium fluorescein. Retinal angiography was immediately performed under dark‐adapted conditions using a fundus imaging system. Post‐procedure, animals were provided with adequate water to facilitate fluorophore clearance.

### 4.12 Real‐Time Quantitative Polymerase Chain Reaction

Total RNA isolation from tissues and cells employed the SteadyPure Mag Tissue and Cells RNA Extraction Kit (Accurate Biotechnology, Hunan, China) following manufacturer standard protocols. Complementary DNA was synthesized for qPCR applications, and quantitative reverse transcription PCR (RT‐qPCR) assays were conducted on an Applied Biosystems 7500 platform (CA, USA) with SYBR Green Premix Pro Taq HS qPCR Kit (Rox Plus; Accurate Biotechnology). The relative gene expression was normalized against β‐actin and calculated via the 2−ΔΔCT method. The sequences of primers used in this study were provided in Table S1.

### 4.13 Western Blotting

Cells and tissues were lysed in RIPA buffer with protease or phosphatase inhibitors. Protein concentrations were measured, and equal amounts were separated by SDS‐PAGE and transferred onto PVDF membranes for analysis. Then, the membranes underwent blocking with 5% non‐fat dried milk for 2–3 h at room temperature. Then, they were incubated with specified primary antibodies for 14–16 h or overnight at 4°C. Subsequently, membranes were incubated with the appropriate secondary antibodies at room temperature for 1 h (Table S2). Protein bands were visualized using chemiluminescent reagents (4A Biotech, China), and signal intensities were quantified with ImageJ software.

### 4.14 Immunofluorescence Assay

Cells cultured in a 24‐well plate for 24–48 h underwent fixation with 4% paraformaldehyde for 20 min. Following fixation, cells were permeabilized with 0.5% Triton X‐100 for 10 min and sealed with 20% goat serum for 1 h at room temperature. Samples were incubated with primary antibodies overnight at 4°C, followed by secondary antibodies for 1 h at room temperature. Fluorescent images were captured using a confocal microscope. For retinal immunofluorescence, the retinas were isolated from the eyeballs and sectioned into a four‐leaf clover to facilitate flat‐mounting under the microscope after fixation with 4% paraformaldehyde for 2 h. Then, retinas were permeabilized with 0.5% Triton X‐100 for 30 min and sealed with 20% goat serum for 1 h at 37°C. For immunolabeling, the tissues were incubated overnight at 4°C with anti‐IBA1 polyclonal antibody (019‐19741; FUJIFILM Wako Pure Chemical Corporation, Japan; 1:500), anti‐CD4 polyclonal antibody (sc‐19641; Santa Cruz Biotechnology,1:100), anti‐F4/80 monoclonal antibody (14‐4801‐85, Thermo Fisher Scientific, 1:50). After three consecutive washes in PBS, the subsequent procedures were performed in accordance with the standard protocol outlined for cellular immunofluorescence.

### 4.15 Reactive Oxygen Species Detection

Simply put, cells were cultured in a 24‐well plate and stimulated with 100 ng/mL LPS for 24 h, then treated with the DCFH‐DA probe or 10 µm dihydroethidium (Solarbio, China) for 40 min at 37°C, and subsequently washed twice with PBS before being detected by fluorescence microscopy.

### 4.16 Intracellular Ca^2^^+^ Measurement

Intracellular calcium levels were measured using the fluorescent calcium indicator Fluo‐4 AM (Beyotime Biotechnology, China) according to the manufacturer's instructions. Briefly, cells were seeded and cultured to approximately 70% confluence, then incubated with Fluo‐4 staining solution at 37°C for 30 min in the dark after washing with PBS. Following incubation, cells were washed three times with PBS and detected by fluorescence microscopy. For calcium chelation experiments, cells were pretreated with the intracellular Ca^2^^+^ chelator BAPTA‐AM (10 µM; MedChemExpress, USA) for one hour prior to incubation.

### 4.17 Enzyme‐Linked Immunosorbent Assay

Levels of inflammatory cytokines or specific proteins in culture supernatants or cell lysates were quantified using commercially available enzyme‐linked immunosorbent assay (ELISA) kits (Elabscience, China) according to the manufacturer's instructions. Briefly, 96‐well plates pre‐coated with capture antibodies were incubated with standards and samples, followed by addition of horseradish peroxidase (HRP)‐conjugated detection antibodies. After color development and termination, the absorbance was measured at 450 nm and analyzed according to the standard.

### 4.18 Transepithelial Electrical Resistance Assay

Cells were seeded onto Transwell inserts (0.4 µm pore size, Corning, USA) and cultured until a confluent monolayer was formed. Transepithelial electrical resistance (TEER) values were measured using an EVOM2 epithelial voltohmmeter with STX2 electrodes at room temperature. Baseline resistance from cell‐free inserts was subtracted from all measurements, and values were normalized to the surface area of the membrane (Ω·cm^2^). Each experimental condition was measured in triplicate, and the mean value was used for subsequent analysis.

### 4.19 Phenol Red Infiltration Assay

The barrier integrity of RPE cell monolayers was assessed using a phenol red infiltration assay. Briefly, 1% phenol red solution in PBS was added to the apical compartment of RPE monolayers cultured on Transwell inserts, following three gentle washes with Hank's balanced salt solution. After incubation at 37°C for 60 min, 100 µL of medium was collected from both the basolateral and apical chambers separately. The absorbance at 560 nm was measured using a microplate reader (Thermo Fisher Scientific, Inc., MA, USA).

### 4.20 Transmission Electron Microscopy

RPE were cultured till tight junctions were established, then cells were initially fixed in 2.5% glutaraldehyde at room temperature for 1 h. The monolayers were then gently detached, transferred to fresh fixative, and maintained at 4°C for an additional 12 h. Samples were dehydrated through graded ethanol, infiltrated with resin, and polymerized. Ultrathin sections were then prepared and stained using standard protocols. Imaging was performed with a Hitachi HT‐7800 transmission electron microscope.

### 4.21 Ethynyl‐2'‐deoxyuridine (EdU) Staining

Briefly, after cells reached quiescence, they were incubated with the EdU (Beyotime, China)working solution. Then, cells were fixed at room temperature with 4% paraformaldehyde solution, and then treated with permeabilization buffer (G1204‐100 ML, Servicebio, China) for 10 min. Click additive solution was incubated at room temperature for 30 min. Nuclei were subsequently stained with 1× Hoechst for 10 min, and images were acquired using a fluorescence microscope (Leica, Germany).

### 4.22 Subretinal Injection of Virus

To ensure effective gene knockdown, subretinal injections were administered to 3‐week‐old mice. The knockdown effect peaked 3–4 weeks after injection and was maintained for more than 2 months thereafter; therefore, EAU was then induced at 6–8 weeks of age (i.e., 3–4 weeks after vitreous injection) to ensure that gene knockdown efficiency at the time of immunization. Subretinal injections were carried out under sterile conditions following refined procedures adapted from established protocols [59, 60]. Mice were anesthetized by intraperitoneal delivery of tribromoethanol, and the ocular surface was desensitized with topical procaine hydrochloride. Under a surgical microscope, an entry site was created at the scleral limbus using a 30‐gauge needle. A 33‐gauge blunt Hamilton needle was subsequently advanced through this entry tract into the subretinal compartment. A total volume of 0.5 µL of adenovirus‐associated virus (titer 10^13^) suspension was infused at a controlled rate, after which the needle was held in place momentarily before withdrawal to prevent reflux. Immediately after injection, antibiotic ointment was applied to the cornea and continued for three days to prevent postoperative infection.

### 4.23 Astral Data Independent Acquisition (DIA) Quantitative Proteomics

Cells were rinsed thoroughly with PBS before harvest. Then cells were prepared with the addition of Dithiothreitol and iodoacetamide, and proteins were subsequently digested using trypsin for 4 h. The resulting peptides were analyzed on a Thermo Orbitrap Astral mass spectrometer to generate raw MS data. DIA‐NN software was used for spectral processing and database searching, retaining only peptides with a Global.Q.Value < 0.01 and proteins with a PG.Q.Value < 0.01. Proteins with a fold change (FC) above or below a defined threshold were considered differentially expressed proteins (DEPs). Functional annotation of identified proteins was performed with InterProScan for Gene Ontology terms and InterPro domain predictions, whereas the Clusters of Orthologous Groups (COG) and Kyoto Encyclopedia of Genes and Genomes (KEGG) databases were applied to classify protein families and map enriched signaling pathways. For differentially expressed proteins (DEPs), volcano plot analysis, hierarchical clustering heatmap analysis, and GO, IPR, and KEGG pathway enrichment analyses were performed. These data were performed at Shanghai Biotree Biomedical Technology Co., Ltd.

### 4.24 RNA‐seq and ChIP‐seq

Cells for RNA‐seq were harvested with PBS washing. Total RNA was isolated and purified using TRIzol reagent (Invitrogen, Carlsbad, CA, USA). After RNA quantity and purity were assessed, Poly(A)+ RNA was enriched from total RNA, fragmented, and reverse‐transcribed, then sequenced on an Illumina NovaSeq 6000 platform to generate 150‐bp paired‐end reads. Raw sequencing reads were quality‐filtered using fastp (https://github.com/OpenGene/fastp) and aligned to the Homo sapiens GRCh38 reference genome using HISAT2 (https://ccb.jhu.edu/software/hisat2). Transcript assembly and expression quantification were performed using StringTie (https://ccb.jhu.edu/software/hisat2), and transcriptomes from all samples were merged using gffcompare. Gene expression levels were calculated as FPKM values. Differential expression analysis was conducted using edgeR (https://bioconductor.org/packages/release/bioc/html/edgeR.html), with fold change > 2 or < 0.5 and p < 0.05 defined as the thresholds for significant differential expression. DAVID (https://david.ncifcrf.gov/) was used for KEGG and GO enrichment analysis.

ChIP assays were performed using the SimpleChIP Plus Sonication Chromatin IP System (Cell Signaling Technology, #56383). ChIP‐seq libraries were prepared using the NEBNext Ultra DNA Library Prep Kit (NEB, USA) and sequenced on an Illumina platform with 150‐bp paired‐end reads. After quality control using FastQC (v0.11.5), clean reads were aligned to the reference genome using Bowtie2 with up to two mismatches allowed. Peaks were identified using MACS2 (v2.1.1) and annotated using HOMER (v4.10). Genome‐wide read distribution and coverage profiles were analyzed using ChIPseeker (v1.5.1) and deepTools (v2.4.1), respectively. Motif enrichment and transcription factor binding analyses were performed using HOMER (v4.10). These data were performed at LC‐Bio Technology CO., Ltd., Hangzhou, China.

### 4.25 Flow Cytometry

Mononuclear cells were isolated from the cervical draining lymph nodes (CDLNs) and spleens of mice. Cells were restimulated with Cell Activation Cocktail containing Brefeldin A (BioLegend,423304) at 37°C for 6 h. After stimulation, cells were stained with Fixable Viability Stain 450 (BD Bioscience, 562247) to label dead cells, followed by surface staining with anti‐mouse CD4 antibody (BioLegend, 100406). Subsequently, cells were fixed, permeabilized, and stained intracellularly with PE/Cyanine7‐conjugated anti‐mouse IFN‐γ (BioLegend, 505825) and PE‐conjugated anti‐mouse IL‐17A (BioLegend, 506903). Stained cell samples were acquired on an Attune NxT flow cytometer (Thermo Fisher Scientific), and raw flow cytometry data were analyzed using FlowJo software (FlowJo Co., Ashland, OR, USA).

### 4.26 Coimmunoprecipitation Assay

Immunoprecipitation was carried out using the Abcam IP Kit (ab206996). Cells were rinsed with PBS and lysed on ice. The supernatants were incubated with anti‐Flag antibody overnight at 4°C with gentle rotation. Protein A/G agarose beads were added the next day to capture immune complexes. The bound proteins were detected by western blotting.

### 4.27 Statistical Analysis

All analyses were performed using SPSS software 20.0 (IBM, USA), and Prism version 8.0 (GraphPad, San Diego, USA). Continuous data are presented as mean ± SD and compared using an unpaired two‐tailed Student's *t*‐test for two groups, or one‐way ANOVA with appropriate post hoc tests for multiple groups. In addition, ordinal data in the scores of EAU model were analyzed using the Kruskal–Wallis test, followed by pairwise Mann–Whitney U tests with Bonferroni correction. All experiments were independently repeated at least three times, and a corrected *p* < 0.05 was considered statistically significant.

## Author Contributions

Q. Z., J. H., Y. W., and X. L. contributed equally to this work. Q.Z., J.H., Y.W., and X.L. designed the study, performed the experiments, and drafted the manuscript. L.D. and X.L. conducted molecular assays. H.L., J.L., Y.D., and Q.W. helped sample collection and WES analysis. G.S., W.Z., Y.D., Q.C., C.H., X.L., Y.L., L.C., and S.H. helped sample collection and provided technical support. P.Y. supervised and designed the study. All authors approved the final manuscript.

## Conflicts of Interest

The author declares no conflicts of interest.

## Supporting information




**Supporting File**: advs76991‐sup‐0001‐SuppMat.docx.

## Data Availability

The sequencing data that support the findings of this study are openly available in NCBI Gene Expression Omnibus (GEO) at https://www.ncbi.nlm.nih.gov/geo/, reference number [GSE334894], [GSE335073] and in iProX at https://www.iprox.cn/, reference number [PXD079501]. The data involving human participants are not publicly available due to privacy and ethical restrictions, but are available on request from the corresponding author upon reasonable request.
