# Supplementary material for: A Rare De Novo Missense Mutation in IFT122 Confers a Genetic Susceptibility Factor of Idiopathic Pediatric Uveitis Via Trio‐based Whole‐Exome Sequencing
Source: Adv Sci (Weinh). 2026 Sep 2:e76991. Online ahead of print. doi: 10.1002/advs.76991 (PMC13537077; doi:10.1002/advs.76991)
Supplement: Supplementary file 1 [file ADVS-9999-e76991-s001.docx]

**Supplementary Fig.1**

**A** IFT122 mRNA expression of peripheral blood in health, PIU patients without and with mutation. (n=3-5/group; mean±SD; NS p>0.05, *p<0.05, **p<0.01; one-way ANOVA).

**Supplementary Fig.2**

**Supplementary Fig.2**

**A** Weight of mice of different ages in wild type (IFT122 ^A773/A773^) and homozygotes (IFT122 ^E773/E773^). (n=4-6/group; mean±SD; NS p>0.05, *p<0.05, **p<0.01; Unpaired Student’s t-test).

B Typical kidney histopathological sections from two groups of mice mentioned above.

**C** Blood urea nitrogen (BUN) of mice in wild type (IFT122 ^A773/A773^) and homozygotes (IFT122 ^E773/E773^) mice (n=5/group; mean±SD; NS p>0.05, *p<0.05, **p<0.01; Unpaired Student's t test).

**D** Creatinine (CREA) of mice in wild type (IFT122 ^A773/A773^) and homozygotes (IFT122 ^E773/E773^) mice. (n=5/group; mean±SD; NS p>0.05, *p<0.05, **p<0.01; Unpaired Student's t test).

**E** Uric acid (UA) of mice in wild type (IFT122 ^A773/A773^) and homozygotes (IFT122 ^E773/E773^) mice. (n=5/group; mean±SD; NS p>0.05, *p<0.05, **p<0.01;Unpaired Student's t test).

**F-H** Anterior segment inflammation, clinical fundus photographs and hematoxylin-eosin staining of the two groups mentioned above.

**I-L** Representative flow cytometry plots and qualification of Th1 and Th17 cells proportions in the spleens and cervical draining lymph nodes (CDLNs) of wild type (IFT122 ^A773/A773^) and homozygotes (IFT122 ^E773/E773^) mice with and without EAU development (n=5/group; mean±SD; NS p>0.05, *p<0.05, **p<0.01; Unpaired Student's t test).

**M** Representative fluorescence images of CD4, IBA1, and F4/80 in the retina of wild type (IFT122 ^A773/A773^) and homozygotes (IFT122 ^E773/E773^) mice with and without EAU development (scale bar, 20μm).

**Supplementary Fig.3**

**A** RPE65-positive cells in primary RPE cells isolated from mice. (scale bar, 100μm).

**B** RPE65-positive cells in primary RPE cells isolated from human. (scale bar, 100μm).

**C** Protein levels and quantitative charts of ift22 of RPE cells isolated from mice before and after EAU development (n=3/group; mean±SD; NS p>0.05, *p<0.05, **p<0.01; Unpaired Student's t test).

**Supplementary Fig.4**

**A** Typical cell morphology under light microscopy in the wild type, negative control, and mutation ARPE19 cells. (scale bar, 100μm)

**B** Typical immunofluorescence appearance of cilia in the wild type, negative control, and mutation ARPE19 cells. (scale bar, 20μm)

**Supplementary Fig.5**

**A** The GO enrichment of DEGs between WT and MUT ARPE19 cells.

**B, C** Protein levels and quantitative charts of p-PKC, p-AKT, JUN, FOS and p-STAT3 in WT, NC and MUT cells (n=4/group; mean±SD; **p<0.01; one-way ANOVA).

**D** The KEGG enrichment of DEGs between WT and MUT ARPE19 cells.

**E, F** Representative fluorescence images of Fluo-4 AM staining in three groups cells (scale bar, 100μm).

**G, H** Protein levels and quantitative charts of MEK, ERK, p-MEK, p-ERK of MUT cells before and after Ca²⁺ chelator BAPTA-AM treatment (n=3/group; mean±SD; NS p>0.05, *p<0.05, **p<0.01; Unpaired Student's t test).

**Supplementary Fig.6**

**A** Heatmap of Differentially Expressed Genes (DEGs) Between between Scram cells (ShNC) and FRA1 knock down (sh-FRA1) cells.

**B** The GO enrichment of DEGs between ShNC and sh-FRA1 cells.

**C** The KEGG enrichment of DEGs between ShNC and sh-FRA1 cells.

**Supplementary Fig.7**

**A** Subretinal injection flowchart.

**B** Fluorescence effect of AAV subretinal injection under fundus examination.

**C** Fluorescence of retinal frozen sections after AAV subretinal injection.

**D** Statistical analysis of AAV injection knockdown effects on the retinal RPE layer.

**Supplementary Table 1**

| Genes | Species | Sequence (5' To 3') |
| --- | --- | --- |
| IFT122-F | HumAn | CCT GGT CAC ATA GTA CAT AGA CC |
| IFT122-R | HumAn | TCT GAA GTA ACT CAA GCA CCT TG |
| IFT122-F | Mouse | GGC TAC AAC GGC TCC AAG AT |
| IFT122-R | Mouse | TCT GTC ACT CCC AAG CAA GC |
| IFT122 knock-in-F | Mouse | TTATTTATAGCCTGCGGCCTGAGA |
| IFT122 knock-in-R | Mouse | GACAAAACACTCAATAAACAAGCCC |
| FRA1-F | HumAn | AGG CAG TAC CAG TGG CAC CAG |
| FRA1-R | HumAn | AAA GGA GTT AGG GAG GGT GTG GTC |
| β-ACTin-F | HumAn | GGATGCAGAAGGAGATCACTG |
| β-ACTin-R | HumAn | CGATCCACACGGAGTACTTG |
| GAPDH-F | Mouse | AGGTCGGTGTGAACGGATTTG |
| GAPDH-R | Mouse | TGTAGACCATGTAGTTGAGGTCA |
| FGF2-F | HumAn | TGCGTCGTCTAATTCAAGATAGGT |
| FGF2-R | HumAn | GGCTGAAGTCGTCTACCAACA |
| ANGPTL6-F | HumAn | TGTGTCTAAATCCAGCCGTGTTA |
| ANGPTL6-R | HumAn | AACTTACTGACTCCTCTCACCAAC |
| MMP14-F | HumAn | GGAATCAAGCCACTCAGAATATGC |
| MMP14-R | HumAn | GCCTGAATCCAATTACAACCAAGA |
| MMP25-F | HumAn | TTCAGATGGCGGTGAGTGTT |
| MMP25R | HumAn | CATGGCTGGTTCCTCCTCTT |
| IL18-F | HumAn | CTTGCTTAGTGTGCTGCTTCTG |
| IL18-R | HumAn | CACCTTCCAACTGTAGAGTCCAA |
| FRA1-F | Mouse | GAAGGACCCAGGTGGTTCTG |
| FRA1-R | Mouse | AGTCAGAGAGGGTGTGGTCA |

**Supplementary Table 2**

| Antibody | Host | Manufacture | Application |
| --- | --- | --- | --- |
| IFT122 | R | Abcam | 1:1000 |
| FLAG | M | Huabio | 1:1000 |
| iNOS | R | Affinity | 1:1000 |
| COX2 | R | Abcam | 1:1000 |
| IL-1β | R | Abcam | 1:1000 |
| CXCL10 | R | Abcam | 1:500 |
| MMP2 | R | Proteintech | 1:1000 |
| MMP9 | R | Proteintech | 1:1000 |
| ZO-1 | M | Abcam | 1:1000 |
| MMP25 | R | Proteintech | 1:500 |
| MMP14 | R | Proteintech | 1:1000 |
| FGF2 | R | Proteintech | 1:1000 |
| p-MEK | R | Cell Signal Technology | 1:1000 |
| MEK | R | Huabio | 1:1000 |
| p-ERK | R | Cell Signal Technology | 1:2000 |
| ERK | R | Cell Signal Technology | 1:2000 |
| p-FRA1 | R | Cell Signal Technology | 1:1000 |
| FRA1 | R | Cell Signal Technology | 1:2000 |
| IFT43 | R | Huabio | 1:500 |
| GAPDH | R | Proteintech | 1:5000 |
| β-actin | R | Proteintech | 1:10000 |
| p-PKC | R | Huabio | 1:1000 |
| CFOS | R | Huabio | 1:1000 |
| p-AKT | R | Huabio | 1:1000 |
| C-JUN | R | Huabio | 1:1000 |
| p-STAT3 | R | Huabio | 1:1000 |
| γ-Tublin | M | Proteintech | 1:1000 |
| ACeTyl-Tublin | R | Proteintech | 1:1000 |
